# Supplementary material for: A hypothesis - generating Swedish extended national cross-sectional family study of multimorbidity severity and venous thromboembolism
Source: BMJ Open. 2023 Jun 16;13(6):e072934. doi: 10.1136/bmjopen-2023-072934 (PMC10277039; doi:10.1136/bmjopen-2023-072934)
Supplement: Supplementary data [file bmjopen-2023-072934supp001.pdf]

Supplementary Appendix: Authors: Ahrén J, Pirouzifard M, Holmquist B, Sundquist J, Halling A, Sundquist K, Zöller B

Table of content pages 1-9 **Pages**

1. Supplementary Table S1 2-3
2. Supplementary Table S2 4
3. Supplementary Table S3 5
4. Supplementary Table S4 6
5. Supplementary Table S5 7
6. Supplementary Table S6 8
7. Supplementary Table S7 9
8. Supplementary Table S8 10

Ahrén J, *et al. BMJ Open* 2023; 13:e072934. doi: 10.1136/bmjopen-2023-072934

Supplementary Table S1. Diseases with ICD codes included in the multimorbidity score modified and adapted to Swedish ICD-10 codes after Barnett K et al (2012)*. Infectious diseases, i.e. viral hepatitis, were excluded and six common non-communicable diseases (NCDs) were added: dermatitis or eczema, gout, arthrosis, osteoporosis, obesity, and pancreatitis. One point for each disease. The theoretical range of the score is 0-45 points.		
	Included disorders in the multimorbidity score	ICD-10**
1	Hypertension	I10-I15
2	Affective disorders	F30-F39
3	Painful back condition	M50-54
4	Asthma	J45-J46
5	CHD	I20-I25
6	Ulcer disease	K221, K25-K28
7	Diabetes	E10-E14
8	Thyroid disorders	E00-E07
9	Connective tissue disease	M05, M06, M08, M09, M30-M36, D86
10	Hearing loss (or impaired)	H90-H91
11	Chronic obstructive pulmonary disease (COPD)	J41-J44
12	Anxiety	F40-F48
13	Irritable bowel syndrome (IBS)	K58
14	Cancer	C00-C99
15	Alcohol use disorders	F10
16	Psychoactive substance misuse	F11-F14, F16, F18, F19
17	Constipation	K590
18	Cerebrovascular disease	I60-I69
19	Renal disease	N00-N19, Q61
20	Diverticular disease of intestine	K57
21	Atrial fibrillation	I48
22	Atherosclerosis	I70-I74, I77
23	Heart failure	I50, I110, I130, I132
24	Prostate disease	N40-N42
25	Glaucoma	H40, H42

Ahrén J, *et al. BMJ Open* 2023; 13:e072934. doi: 10.1136/bmjopen-2023-072934

26	Epilepsy	G40, G41
27	Dementia	F00-F03
28	Schizophrenia disorders	F20, F21
29	Psoriasis	L40
30	Dermatitis or Eczema	L20-L30
31	Inflammatory bowel disease (IBD)	K50, K51
32	Migraine	G43, G440, G441
33	Blindness & low vision	H53-H54
34	Chronic sinusitis	J32
35	Learning disability	F81
36	Anorexia or bulimia	F50
37	Bronchiectasis	J47
38	Parkinson's disease	G20, G21, G22
39	Multiple sclerosis	G35
40	Liver disease	K70-K77
41	Gout	M10
42	Arthrosis	M15-M19
43	Osteoporosis	M80-M82
44	Obesity	E65, E66
45	Pancreas diseases	K85, K86

*Barnett K, Mercer SW, Norbury M, Watt G, Wyke S, Guthrie B. Epidemiology of multimorbidity and implications for health care, research, and medical education: a cross-sectional study. Lancet 2012; 380: 37-43.

**ICD=International classification of disease.

Supplementary Table 2. Descriptive findings for all unique study participants with stratification according to multimorbidity scores for number of unique individuals, sex, education, age at the end of study, birth date, and VTE (venous thromboembolism).

		Multimorbidity scores							
	All	0	1	2	3	4	≥ 5	≤ 1	≥ 2
Unique individuals, n (%)	2694442 (100)	1616213 (59.98)	637487 (23.66)	254210 (9.43)	104634 (3.88)	45585 (1.69)	36313 (1.35)	2253700 (83.64)	440742 (16.36)
Sex, Female, % (n)	48.73 (1312989)	45.25 (731337)	51.41 (327721)	55.66 (141488)	59.07 (61808)	60.68 (27661)	63.27 (22974)	46.99 (1059058)	57.61 (253931)
Education (≥12 years), % (n)	30.36 (818146)	31.97 (516774)	29.67 (189127)	27.16 (69050)	24.80 (25946)	22.45 (10232)	19.32 (7017)	31.32 (705901)	25.47 (112245)
Age at end of study, Median (IQR) Range (min-max)	32 (22-43) (0 -68)	31 (22-42) (0-67)	32 (22-43) (0-67)	33 (23-45) (0-67)	36 (25-46) (1-66)	38 (27-48) (1-67)	42 (31-50) (3-68)	31 (22-42) (0-67)	35 (24-46) (0-68)
Year of birth, Median (IQR) Range (min-max)	1983 (1972-1993) (1947-2005)	1984 (1973-1993) (1948-2005)	1983 (1972-1993) (1948-2005)	1981 (1970-1992) (1948-2005)	1979 (1968-1990) (1949-2005)	1977 (1967-1988) (1948-2005)	1972 (1964-1984) (1947-2005)	1984 (1973-1993) (1948-2005)	1980 (1969-1990) (1947-2005)
VTE, n (%)	16099 (0.6)	5008 (0.31)	4108 (0.64)	2652 (1.04)	1665 (1.59)	1063 (2.33)	1603 (4.41)	9116 (0.40)	6983 (1.58)

IQR=Interquartile Range.

Supplementary Table S3. Odds ratio for VTE according to multimorbidity score (0 to ≥5) in males. Odds Ratios (ORs) with 95 % confidence interval (CI) for multimorbidity scores. Reference with no diseases (score=0).

	Male (n=1381453)			
	OR (95% CI)			
VTE score	No VTE	VTE	Model 1	Model 2
Score 0	882367	2509	1[Reference]	1[Reference]
Score 1	307866	1900	2.17 (2.04-2.30)	2.03 (1.91-2.26)
Score 2	111533	1189	3.75 (3.5-4.02)	3.20 (2.99-3.44)
Score 3	42063	763	6.38 (5.88-6.92)	4.73 (4.35-5.14)
Score 4	17441	483	9.74 (8.83-10.75)	6.3(5.69-6.96)
Score ≥5	12695	644	17.84 (16.33-19.49)	9.38 (8.55-10.28)

Abbreviations: OR, odds ratio. Model 1 is a crude model (univariable). Model 2 is an adjusted model (multivariable), with adjustments for year of birth, county, and educational attainment.

Ahrén J, *et al. BMJ Open* 2023; 13:e072934. doi: 10.1136/bmjopen-2023-072934

Supplementary Table S4. Odds ratio for VTE according to multimorbidity score (0 to ≥5) in females. Odds Ratios (ORs) with 95 % confidence interval (CI) for multimorbidity scores. Reference with no diseases (score=0).

	Female (n=1312989)			
	OR (95% CI)			
VTE score	No VTE	VTE	Model 1	Model 2
Score 0	728838	2499	1[Reference]	1[Reference]
Score 1	325513	2208	1.98 (1.88-2.10)	1.84 (1.73-1.94)
Score 2	140025	1463	3.05 (2.86-3.25)	2.67 (2.5-2.85)
Score 3	60906	902	4.32 (4.0-4.66)	3.53 (3.26-3.81)
Score 4	27081	580	6.25 (5.70-6.84)	4.76 (4.34-5.22)
Score ≥5	22015	959	12.70 (11.78-13.70)	8.58 (7.94-9.27)

Abbreviations: OR, odds ratio. Model 1 is a crude model (univariable). Model 2 is an adjusted model (multivariable), with adjustments for year of birth, county, and educational attainment.

Ahrén J, *et al. BMJ Open* 2023; 13:e072934. doi: 10.1136/bmjopen-2023-072934

Supplementary Table S5. Odds ratio for VTE according to multimorbidity score (0 to ≥5) for individuals born 1947-1972. Odds Ratios (ORs) with 95 % confidence interval (CI) for multimorbidity scores. Reference with no diseases (score=0).

	All (n=641211)			
	OR (95% CI)			
VTE score	NO VTE	VTE	Model 1	Model 2
Score 0	340353	2331	1[Reference]	1[Reference]
Score 1	156444	2062	1.92 (1.78-2.00)	1.89 (1.78-2.00)
Score 2	70005	1437	3 (2.81-3.2)	2.89 (2.7-3.08)
Score 3	33176	957	4.22 (3.9-4.54)	4 (3.7-4.31)
Score 4	16542	624	5.51 (5.04-6.02)	5.13 (4.68-5.62)
Score ≥5	16266	1014	9.1 (8.44-9.81)	8.3 (7.68-8.97)

Abbreviations: OR, odds ratio. Model 1 is a crude model (univariable). Model 2 is an adjusted model (multivariable), with adjustments for sex, year of birth, county, and educational attainment.

Ahrén J, *et al. BMJ Open* 2023; 13:e072934. doi: 10.1136/bmjopen-2023-072934

Supplementary Table S6. Odds ratio for VTE according to multimorbidity score (0 to ≥5) for individuals born 1972-1983. Odds Ratios (ORs) with 95 % confidence interval (CI) for multimorbidity scores. Reference with no diseases (score=0).

	All (n=682582)			
	OR (95% CI)			
VTE score	NO VTE	VTE	Model 1	Model 2
Score 0	415987	1638	1[Reference]	1[Reference]
Score 1	155822	1277	2.08 (1.93-2.24)	2.01 (1.87-2.16)
Score 2	60801	725	3.02 (2.77-3.31)	2.85 (2.61-3.11)
Score 3	25237	468	4.71 (4.25-5.22)	4.33 (3.9-4.81)
Score 4	11184	280	6.36 (5.59-7.23)	5.74 (5.04-6.54)
Score ≥5	8779	384	11.11 (9.92-12.44)	9.7 (8.64-10.89)

Abbreviations: OR, odds ratio. Model 1 is a crude model (univariable). Model 2 is an adjusted model (multivariable), with adjustments for sex, year of birth, county, and educational attainment.

Supplementary Table S7. Odds ratio for VTE according to multimorbidity score (0 to ≥5) for individuals born 1983-1993. Odds Ratios (ORs) with 95 % confidence interval (CI) for multimorbidity scores. Reference with no diseases (score=0).				
	All (n=691267)			
	OR (95% CI)			
VTE score	NO VTE	VTE	Model 1	Model 2
Score 0	427804	845	1[Reference]	1[Reference]
Score 1	156178	624	2.02 (1.82-2.24)	1.94 (1.75-2.15)
Score 2	61537	399	3.28 (2.91-3.7)	3.04 (2.69-3.43)
Score 3	25768	204	4.01 (3.44-4.67)	3.59 (3.07-4.2)
Score 4	10734	137	6.46 (5.39-7.75)	5.63 (4.68-6.78)
Score ≥5	6858	179	13.21 (11.23-15.55)	10.91 (9.21-12.91)
Abbreviations: OR, odds ratio. Model 1 is a crude model (univariable). Model 2 is an adjusted model (multivariable), with adjustments for sex, year of birth, county, and educational attainment.				

Supplementary Table S8. Odds ratio for VTE according to multimorbidity score (0 to ≥5) for individuals born 1993-2005. Odds Ratios (ORs) with 95 % confidence interval (CI) for multimorbidity scores. Reference with no diseases (score=0).

	All<br>(n=679382)			
	OR (95% CI)			
VTE score	NO VTE	VTE	Model 1	Model 2
Score 0	427061	194	1[Reference]	1[Reference]
Score 1	164935	145	1.94 (1.56-2.4)	1.94 (1.56-2.41)
Score 2	59215	91	3.38 (2.64-4.34)	3.16 (2.46-4.06)
Score 3	18788	36	4.22 (2.95-6.02)	3.49 (2.44-5)
Score 4	6062	22	7.99 (5.14-12.43)	5.92 (3.8-9.24)
Score ≥5	2807	26	20.4 (13.52-30.76)	13.46 (8.87-20.42)

Abbreviations: OR, odds ratio. Model 1 is a crude model (univariable). Model 2 is an adjusted model (multivariable), with adjustments for sex, year of birth, county, and educational attainment.
