# Supplementary material for: A hypothesis - generating Swedish extended national cross-sectional family study of multimorbidity severity and venous thromboembolism
Source: BMJ Open. 2023 Jun 16;13(6):e072934. doi: 10.1136/bmjopen-2023-072934 (PMC10277039; doi:10.1136/bmjopen-2023-072934)
Supplement: Author's manuscript [file bmjopen-2023-072934.draft_revisions.pdf]

BMJ Open

BMJ Open is committed to open peer review. As part of this commitment we make the peer review history of every article we publish publicly available.

When an article is published we post the peer reviewers' comments and the authors' responses online. We also post the versions of the paper that were used during peer review. These are the versions that the peer review comments apply to.

The versions of the paper that follow are the versions that were submitted during the peer review process. They are not the versions of record or the final published versions. They should not be cited or distributed as the published version of this manuscript.

BMJ Open is an open access journal and the full, final, typeset and author-corrected version of record of the manuscript is available on our site with no access controls, subscription charges or pay-per-view fees (http://bmjopen.bmj.com).

If you have any questions on BMJ Open's open peer review process please email info.bmjopen@bmj.com

BMJ Open

Association between multimorbidity severity and odds for venous thromboembolism

Journal:	*BMJ Open*
Manuscript ID	bmjopen-2023-072934
Article Type:	Original research
Date Submitted by the Author:	17-Feb-2023
Complete List of Authors:	Ahrén, Jonatan; Lund University, Center for Primary Health Care Research<br>Pirouzifard, Mirnabi; Lund University, Center for Primary Health Care Research<br>Holmquist, Björn; Lund University Department of Statistics, Department of Statistics<br>Sundquist, Jan; Lund University, Center for Primary Health Care Research<br>Halling, Anders; Lunds Universitet, Center for Primary Health Care Research<br>Sundquist, Kristina; Lund University, Center for Primary Health Care Research<br>Zöller, Bengt; Lund University, Center for Primary Health Care Research
Keywords:	Thromboembolism < CARDIOLOGY, EPIDEMIOLOGY, GENERAL MEDICINE (see Internal Medicine), Vascular medicine < INTERNAL MEDICINE, Primary Health Care

SCHOLARONE™
Manuscripts

BMJ

I, the Submitting Author has the right to grant and does grant on behalf of all authors of the Work (as defined in the below author licence), an exclusive licence and/or a non-exclusive licence for contributions from authors who are: i) UK Crown employees; ii) where BMJ has agreed a CC-BY licence shall apply, and/or iii) in accordance with the terms applicable for US Federal Government officers or employees acting as part of their official duties; on a worldwide, perpetual, irrevocable, royalty-free basis to BMJ Publishing Group Ltd ("BMJ") its licensees and where the relevant Journal is co-owned by BMJ to the co-owners of the Journal, to publish the Work in this journal and any other BMJ products and to exploit all rights, as set out in our licence.

The Submitting Author accepts and understands that any supply made under these terms is made by BMJ to the Submitting Author unless you are acting as an employee on behalf of your employer or a postgraduate student of an affiliated institution which is paying any applicable article publishing charge ("APC") for Open Access articles. Where the Submitting Author wishes to make the Work available on an Open Access basis (and intends to pay the relevant APC), the terms of reuse of such Open Access shall be governed by a Creative Commons licence – details of these licences and which Creative Commons licence will apply to this Work are set out in our licence referred to above.

Other than as permitted in any relevant BMJ Author's Self Archiving Policies, I confirm this Work has not been accepted for publication elsewhere, is not being considered for publication elsewhere and does not duplicate material already published. I confirm all authors consent to publication of this Work and authorise the granting of this licence.

For peer review only

Association between multimorbidity severity and odds for venous thromboembolism

[1]Jonatan Ahrén MD, [1]MirNabi Pirouzifard, PhD; [2]Björn Holmquist, PhD; [1]Jan Sundquist, MD, PhD; [1]Anders Halling PhD, MD; [1]Kristina Sundquist, MD, PhD, [1]Bengt Zöller MD, PhD.

[1]Center for Primary Health Care Research, Lund University/Region Skåne, Malmö, Sweden.

[2]Department of Statistics Lund University Lund Sweden

For peer review only

Running Title: Multimorbidity and venous thromboembolism

Correspondence:

Dr Jonatan Ahrén

Center for Primary Health Care Research

CRC, Building 28, Floor 11

Jan Waldenströms gata 35

Skåne University Hospital

S-205 02 Malmö, Sweden

Tel: +46 735127050; Fax: +46 40 391370

jonatan.ahren@med.lu.se

Word count: 3054 **Abstract word count:** 299

Keywords: venous thromboembolism, multimorbidity, epidemiology, medicine, public health, genetics medical

For peer review only - http://bmjopen.bmj.com/site/about/guidelines.xhtml

Summary boxes

What is already known on this topic:

- Venous thromboembolism (VTE) is a common worldwide disease.

- The burden of multimorbidity, defined as, two or more chronic diseases, has increased.

- This study aimed to see if there is an association between multimorbidity and venous thromboembolism.

What this study adds:

- Increasing multimorbidity exhibits an increased association with VTE.

- Familial associations indicate a possible genetic contribution.

- The strong association between multimorbidity and VTE suggests that multimorbidity is a new risk factor for VTE that is of clinical importance.

How this study might affect research, practice or policy:

- Further studies need to be done to map which disease combinations in the multimorbid patient that contribute most to the increased risk of VTE.

- Further studies need to be done to develop tools to distinguish which patients may have use of prophylactic treatment.

For peer review only - http://bmjopen.bmj.com/site/about/guidelines.xhtml

Abstract

Objectives: Venous thromboembolism (VTE) is a common worldwide disease. The burden of multimorbidity, i.e. two or more chronic diseases, has increased. Whether multimorbidity increases VTE risk is a subject that remains to be studied. Our aim was to determine any association between multimorbidity and VTE and any possible familial aggregation.

Design: A nationwide cross-sectional family study between 1997-2015.

Setting: The Swedish Multi-Generation Register, the National Patient Register, the Total Population Register and the Swedish cause of death register were linked.

Participants: 2,694,442 unique individuals were analyzed for VTE and multimorbidity.

Main Outcomes and Measures: Multimorbidity was determined by a counting method using 45 non-communicable diseases (NCD). Multimorbidity was defined by the occurrence of ≥2 diseases. A multimorbidity score was constructed defined by, 0, 1, 2, 3, 4 or 5 or more diseases.

Results: Sixteen percent (n=440742) of the study population was multimorbid. Of the multimorbid patients 58 % were females. There was an association between multimorbidity and VTE. The adjusted odds ratio (OR) for VTE in individuals with multimorbidity (2≥diagnoses) was 3.16 (95%CI 3.06-3.27) compared to individuals without multimorbidity. There was an association between number of diseases and VTE. The adjusted OR was 1.94 (95%CI 1.86-2.02) for one disease, 2.93 (95%CI 2.80-3.08) for two diseases, 4.07 (95%CI 3.85-4.31) for three diseases, 5.46 (95%CI 5.10-5.85) for four diseases, and 9.08 (95%CI 8.56-9.64) for five or more diseases. The association between multimorbidity and VTE was stronger in males OR 3.45 (3.29-3.62) than in females OR 2.91 (2.77-3.04). There were significant but mostly weak familial associations between multimorbidity in relatives and VTE.

For peer review only

For peer review only - http://bmjopen.bmj.com/site/about/guidelines.xhtml

Conclusions: Increasing multimorbidity exhibits a strong and increasing association with VTE. Familial associations indicate a possible genetic contribution. The strong association between multimorbidity and VTE suggests that multimorbidity is a risk factor for VTE that is of clinical importance.

For peer review only

For peer review only - http://bmjopen.bmj.com/site/about/guidelines.xhtml

Strength and limitations of this study

- High coverage and high validity of the registers and the large number of unique individuals.

- Only Swedish-born individuals born by Swedish parents were included. It therefore remains for a future study to show if the results may be generalized to other populations.

- The data did not include biological and environmental risk factors, to make up for the absence of such data we adjusted for educational level, which is related to lifestyle factors.

For peer review only

Introduction

Venous thromboembolism (VTE) is the third most common cardiovascular disease with an incidence of around 1-3 per 1000 individuals.[1-4] The incidence rate increases with age. VTE is rare among children with an incidence of approximately 1 per 100000 and common among elderly people >70 years and above with an incidence of around 1% per year.[5] The lifetime risk of VTE could be up to 10% in certain countries, for instance in Sweden.[3,4] VTE usually manifests as deep venous thrombosis (DVT) of the lower extremities or as pulmonary embolism (PE).[6] The consequences of VTE range from post-thrombotic syndrome, which affects approximately 20% of patients with DVT, to acute death in 1-2% of PE patients.[7,8] The 90-day mortality after VTE is around 5%.[9]

Risk factors for VTE are either acquired or genetic but they often interact.[8] Among the genetic risk factors, the most prominent are the classical thrombophilia, i.e. factor V Leiden (rs6025), the prothrombin G20210A mutation (rs1799963), deficiencies of antithrombin, protein C and protein S.[10,11] Among the acquired risk factors, age is the most significant with a steeply increased risk with advanced age.[5,6] Other acquired risk factors are for instance immobilization, trauma and surgery, malignancy, pregnancy and puerperium, oral contraception, and autoimmune disorders.[1,2,6,12,13]

Risk factors for VTE may work alone or in concert.[6,12] Multimorbidity, i.e. the presence of two or more diseases, increases with age.[14] As patients get older and the field of medicine advances the patients more often present with multiple diseases - multimorbidity.[14] However, multimorbidity also occurs among younger persons.[15] Due to this there is a need to investigate multimorbidity as a risk factor for additional illnesses, such as VTE. With the future

For peer review only - http://bmjopen.bmj.com/site/about/guidelines.xhtml

demographic change with an increasingly elderly population with multimorbidity, there may be a need to adopt a multi-disease framework for clinical diagnostics and treatment. Despite the need to characterize multimorbidity there is no established definition of which morbidities should be included to define multimorbidity.[16] Multimorbidity has often been defined as two or more chronic non-communicable diseases (NCD).[16,17] Barnett et al used 40 diseases in a Scottish cross-sectional study and defined multimorbidity as the presence of two or more diseases where the prevalence of multimorbidity was 23.2% and was present in most people 65 years of age and older.[14] Although the frequency of multimorbidity increases with age, the burden of multimorbidity may be more common in those aged 65 years or younger, and this is especially common in socially deprived neighborhoods.[18]

We aimed to determine the impact of multimorbidity on VTE. We used large Swedish national registers to study the association between multimorbidity and VTE. A modified list of 45 NCDs diagnoses used by Barnett et al was used to define multimorbidity as the presence of two or more of 45 NCDs.[14] To determine a possible association between multimorbidity and VTE we also examined the association between the number of diseases and VTE. We also investigated whether familial association of multimorbidity and VTE exist. Familial association of a disease is a necessary, though insufficient, condition to infer the importance of genetic susceptibility.[19]

For peer review only - http://bmjopen.bmj.com/site/about/guidelines.xhtml

Material and methods

Registers

The following registers were used in the study: The Swedish Multi-Generation Register contains data of familial relationships; the National Patient Register (NPR) contains hospital discharge diagnoses between 1964-2015 with nationwide coverage from 1987 and from 2001-2015 outpatient hospital diagnoses; the Total Population Register with data on death date, education, marital status, migration; and the Swedish cause of death register.[20-25] We used the unique Swedish personal identity number (PIN) to link the databases. The PIN was, however, replaced with a serial number to ensure confidentiality by Statistics Sweden. The validity of Swedish registers has been investigated previously and they have a high degree of coverage and validity.[20-25] Statistics Sweden and the National Board of Health and Welfare provided the register data to us.

Study design

A nationwide cross-sectional study between 1997-2015. This period was chosen to increase the coverage of the registers and to use only the latest versions of the international classification of disease (ICD) 10 codes. To minimize the risk of an incorrect PIN only Swedish-born individuals were included.[21] To investigate any possible familial aggregation we included pairs of full siblings born between 1948 and 2005 by Swedish parents that could further be linked to their Swedish-born half-siblings, and cousins to minimize this source of error. Parents in the study were born between 1932 and 1985 in Sweden. The Swedish National Multi-Generation Register was used to link relatives. We excluded the families with members who died or emigrated before 1997 or emigrated before the age of 17. Both biological parents were obligatorily known, i.e. pedigrees with two parents and two children.

For peer review only - http://bmjopen.bmj.com/site/about/guidelines.xhtml

Full siblings were thereafter linked with related families and with the full siblings' half-siblings and cousins. Full siblings will hereafter be referred to as “siblings”. One dataset with all individuals entered only once was created. Four datasets for familial relations were created: twins, siblings, half-siblings, and cousins. In the four familial datasets, all relative pairs were double-entered, i.e. all sibling pairs, all twins’ pairs, all half-sibling pairs, and all cousin pairs, as described previously.[26] The double entry approach is a common procedure in genetics.[26] Zygosity was assessed only from sex, i.e. twins with different sex were considered to be DZ twins. We had no access to zygosity data. We allowed the same person to be included in more than one family relationship.

Definition of venous thromboembolism

Cases of VTE (i.e., affection status) were classified according to the World Health Organization International Classification of Diseases, Tenth Revision (ICD-10). VTE was defined using the hospital discharge register, main and supplementary diagnoses (between 1997 and 2015) by the following ICD codes: ICD-10: I80 (not I80.0) and I26.[11] It was also defined in the Outpatient Care Register (between 2001 and 2015) by the same ICD-10 codes as above and the cause of death register. Thus, VTE included pulmonary embolism and deep vein thrombosis of the lower extremities. The diagnosis of VTE in the NPR has been shown to have an accuracy of 95%, whereas the overall validity of the NPR is 87%.[22,27] A quality control of the NPR register was performed for 118 patients with VTE in the Malmö diet and cancer study cohort. In 106 (90%) of 118 cases the diagnosis of VTE was correct.[4]

For peer review only - http://bmjopen.bmj.com/site/about/guidelines.xhtml

Multimorbidity score

Since there is no clear definition of which NCDs should be included, the NCDs used in Barnett et al. were used and modified to adapt to the Swedish ICD-10 codes (Supplementary Table S1). Further modifications were made to the list and infectious diseases were excluded. Five other clinically important and common NCDs contributing to disease burden were added instead: pancreatitis, obesity, gout, arthrosis, and osteoporosis. Furthermore, psoriasis and dermatitis or eczema were counted separately. These modifications were made to increase the number of NCDs to get a more comprehensive list. Thus, a total of 45 diagnoses were included and an individual with two or more NCDs was considered to have multimorbidity. Each diagnosis in an individual during the study period was awarded one point per disease and was cumulatively counted. An individual could theoretically have 45 points. In the study, no individual obtained more than 20 points.

Statistical analysis

Logistic regression was used to investigate the association between VTE and multimorbidity. Multimorbidity was defined as two or more diseases. Multimorbidity was also investigated as a cumulative score in order to determine any possible increasing association. Results are presented as odds ratios (ORs) with 95% confidence intervals (95%CI). Adjustments were made for year of birth, sex, region at birth and level of education. Stratified analysis was performed according to sex and birth year quartiles. The familial associations were determined using the double-entry approach.[26] The ambiguity in unselected samples as to which twin's, sibling's, half-sibling's or cousin's trait should be used as the dependent, and which as the independent variable, could be resolved by using double-entry.[26] Each twin, sibling, half-sibling, or cousin is entered twice in the data, and each member of a twin, sibling,

For peer review only - http://bmjopen.bmj.com/site/about/guidelines.xhtml

half-sibling, or cousin pair provides once the dependent and once the explanatory variable.[26] While the consistency of the regression estimates for heritability and environmental influences is not affected by double-entry, the standard errors of the coefficients are biased and need to be adjusted.[26] In the present study in the logistic regression models, we used the "variance covariance (vce) cluster" method using STATA, which calculated robust standard errors using families as clusters.[28] The vce (cluster clustvar) specifies that the standard errors allow for intragroup correlation, relaxing the usual requirement that the observations be independent.

Statistical significance was set to $p<0,05$, all tests were 2-tailed. Data were analyzed using STATA version 16.1 (StataCorp LLC, College Station, TX, USA)

Ethical approval

This study was approved by the Regional Ethical Review Board in Lund (2012/795 and later amendments).

For peer review only

For peer review only - http://bmjopen.bmj.com/site/about/guidelines.xhtml

Results

Multimorbidity

A total of 2,694,442 unique individuals were included in the study, 49% of these were females. The mean age at the end of the study was 32 years. During the study period between 1997 and 2015, a total of 440,742 (16.6%) individuals were diagnosed with multimorbidity (Table 1). Of these 253,931 (58%) were females and the proportion of females increased with increasing multimorbidity score with 63% of the individuals with a score of ≥5 being female. Multimorbidity was associated with level of education where 25% of the multimorbid individuals had an education of ≥12 years compared to 31% in the non-multimorbid group (Table 1). Multimorbidity also increased with older age at the end of the study, multimorbid individuals had a mean age of 35 years versus 31 in non-multimorbid groups. Baseline characteristics can be found in Table 1.

Table 1. Descriptive findings for all unique study participants with stratification according to multimorbidity scores for number of unique individuals, sex, education, age at the end of study, birth date, and VTE (venous thromboembolism).

		Multimorbidity scores							
	All	0	1	2	3	4	≥ 5	≤ 1	≥ 2
Unique individuals, n (%)	2694442	1616213 (59.98)	637487 (23.66)	254210 (9.43)	104634 (3.88)	45585 (1.69)	36313 (1.35)	2253700 (83.64)	440742 (16.36)
Sex, Female, n (%)	1312989 (48.73)	731337 (45.25)	327721 (51.41)	141488 (55.66)	61808 (59.07)	27661 (60.68)	22974 (63.27)	1059058 (46.99)	253931 (57.61)
Education (≥12 years), n (%)	818146 (30.36)	516774 (31.97)	189127 (29.67)	69050 (27.16)	25946 (24.80)	10232 (22.45)	7017 (19.32)	705901 (31.32)	112245 (25.47)
Age at end of study, Median (IQR) Range (min-max)	32 (22-43) (0 -68)	31 (22-42) (0-67)	32 (22-43) (0-67)	33 (23-45) (0-67)	36 (25-46) (1-66)	38 (27-48) (1-67)	42 (31-50) (3-68)	31 (22-42) (0-67)	35 (24-46) (0-68)
Year of birth, Median (IQR) Range (min-max)	1983 (1972-1993) (1947-2005)	1984 (1973-1993) (1948-2005)	1983 (1972-1993) (1948-2005)	1981 (1970-1992) (1948-2005)	1979 (1968-1990) (1949-2005)	1977 (1967-1988) (1948-2005)	1972 (1964-1984) (1947-2005)	1984 (1973-1993) (1948-2005)	1980 (1969-1990) (1947-2005)
VTE, n (%)	16099 (0.6)	5008 (0.31)	4108 (0.64)	2652 (1.04)	1665 (1.59)	1063 (2.33)	1603 (4.41)	9116 (0.40)	6983 (1.58)

IQR=Interquartile Range.

For peer review only - http://bmjopen.bmj.com/site/about/guidelines.xhtml

VTE

There was a total of 16,099 cases with diagnosed VTE during the study period between 1997 and 2015. In individuals with multimorbidity, 1.58 % had VTE compared to 0.4% in individuals without multimorbidity. Of the female individuals 0.66% had VTE and among males the figure was 0.54%. VTE increased with age with 1.3% in the oldest group and 0.08% in the youngest group. (Tables 1-3).

Table 2. Odds ratio (OR) for VTE (venous thromboembolism) and multimorbidity (score ≥2) for all and stratified according to sex. Odds Ratios (ORs) with 95 % confidence interval (CI) for multimorbidity. Reference with one or no diseases (score=0 or 1). Total observations n=2694442.

	OR (95% CI)			
	No VTE	VTE	Model 1	Model 2
All	433759	6983	3.96 (3.84-4.09)	3.16 (3.06-3.27)
Males	183732	3079	4.52 (4.32-4.74)	3.45 (3.29-3.62)
Females	250027	3904	3.5 (3.35-3.65)	2.91 (2.79-3.04)

Abbreviations: OR, odds ratio. Model 1 is a crude model (univariate). Model 2 is an adjusted model (multivariate), with adjustments for year of birth, county, and educational attainment.

Table 3. Odds ratio for VTE (venous thromboembolism) according to multimorbidity score (0 to ≥2) and age stratified according to interquartile range (IQR1-4). Odds Ratios (ORs) with 95 % confidence interval (CI) for multimorbidity scores. Reference with no diseases (score=0). Total observations n=2694442.

	OR (95% CI)			
Age groups	No VTE	VTE	Model 1	Model 2
IQR1	135989	4032	3.35 (3.21-3.50)	3.14 (3.01-3.29)
IQR2	106001	1857	3.43 (3.24-3.64)	3.14 (2.96-3.34)
IQR3	104897	919	3.48 (3.21-3.78)	3.09 (2.84-3.37)
IQR4	86872	175	3.52 (2.93-4.22)	3.07 (2.56-3.7)

Abbreviations: OR, odds ratio. Model 1 is a crude model (univariate). Model 2 is an adjusted model (multivariate), with adjustments for sex, year of birth, county, and educational attainment. IQR=interquartile range. IQR1 1947-1972, IQR2 1972-1983, IQR3 1983-1993, IQR4 1993-2005.

For peer review only - http://bmjopen.bmj.com/site/about/guidelines.xhtml

VTE and multimorbidity

In the multimorbidity group a total of 6983 of the 440742 (1.58%) individuals were diagnosed with VTE (Table 1). In the group with a multimorbidity score of ≥5 1603 of the 36313 (4.41%) had VTE during the study period (Table 1). The OR for VTE in the adjusted model for an individual with multimorbidity was 3.16 (95%CI 3.06 to 3.27) (Table 2). For males with multimorbidity, the OR for VTE was higher than for females (Table 2). Birth year was stratified into quartiles. For individuals born 1947-1972 the OR for VTE with multimorbidity was 3.14 (95%CI: 3.01 to 3.29) in the adjusted model and for individuals born 1993-2005 the OR for VTE with multimorbidity was 3.07 (95%CI 2.56 to 3.7) (Table 3). Thus, there was no strong age dependence regarding the association between multimorbidity and VTE.

Association between increasing multimorbidity score and VTE

The association of increasing multimorbidity score on VTE was investigated (Table 4). It showed an increased OR for increasing score of multimorbidity. OR for a multimorbidity score of two in the adjusted model was 2.93 (95%CI 2.80 to 3.08), 4.07 (95%CI 3.85 to 4.31) for a multimorbidity score of three, 5.46 (95%CI: 5.10 to 5.85) for a multimorbidity score of four, and for a multimorbidity score of five or more the OR was 9.08 (95%CI: 8.56 to 9.64). (Table 4).

Table 4. Odds ratio for VTE (venous thromboembolism) according to multimorbidity score (0 to ≥5). Odds Ratios (ORs) with 95 % confidence interval (CI) for multimorbidity scores. Reference with no diseases (score=0).

	All (n=2694442)			
	OR (95% CI)			
Multimorbidity score	No VTE	VTE	Model 1	Model 2
Score 0	1611205	5008	1[Reference]	1[Reference]
Score 1	633379	4108	2.09 (2.00-2.18)	1.94 (1.86-2.02)

For peer review only - http://bmjopen.bmj.com/site/about/guidelines.xhtml

Score 2	251558	2652	3.39 (3.24-3.56)	2.93 (2.80-3.08)
Score 3	102969	1665	5.20 (4.92-5.50)	4.07 (3.85-4.31)
Score 4	44522	1063	7.68 (7.18-8.21)	5.46(5.10-5.85)
Score ≥5	34710	1603	14.86 (14.03-15.73)	9.08 (8.56-9.64)

Abbreviations: OR, odds ratio. Model 1 is a crude model (univariate). Model 2 is an adjusted model (multivariate), with adjustments for sex, year of birth, county, and educational attainment.

Age and sex stratification

Multimorbidity score results for sex and age stratification are presented in Supplementary Tables S2-S7. A strong association between multimorbidity and VTE could be observed in both sexes and all age groups.

Familial associations between multimorbidity and VTE

There was mostly a weak association between twin, sibling, half-sibling and cousin history of multimorbidity and VTE (Table 5). The ORs in adjusted models for VTE in patients with multimorbidity were 1.89 in twins (95%CI 1.17 to 3.05), 1.18 in siblings (95%CI 1.12 to 1.24), 1.1 in half-siblings (95%CI 1.03 to 1.18), and 1.07 in cousins (95%CI 1.04 to 1.1) of

For peer review only

For peer review only - http://bmjopen.bmj.com/site/about/guidelines.xhtml

those diagnosed with index score more than one (Table 5). Among twins, the adjusted OR for VTE in patients with multimorbidity was =0.73 (95%CI 0.04 to 1.35) for twin score=1, OR=1.57 (95%CI 0.81 to 3.05) for twin score=2, OR=1.26 (95%CI 0.48 to 3.31) for twin score=3, OR=1.48 (95%CI 0.44 to 4.98) for twin score=4, and OR=3.98 (95%CI 1.17 to 9.39) for twin score ≥ 5.

For peer review only

Table 5. Familial associations for multimorbidity and venous thromboembolism (VTE). Odds ratio with 95 % confidence interval (CI) for VTE according to multimorbidity (≥ 2 score) or multimorbidity score (0 to ≥5) in relatives. Reference are relatives with no diseases (score=0) for score but no or one disease for multimorbidity (≥ 2 score).

	Twins	Siblings	Half-siblings	Cousins

For peer review only - http://bmjopen.bmj.com/site/about/guidelines.xhtml

	(n=24020)		(n=1546108)		(n=984976)		(n=6623156)	
	OR (95% CI)		OR (95% CI)		OR (95% CI)		OR (95% CI)	
Relative score	Model 1	Model 2	Model 1	Model 2	Model 1	Model 2	Model 1	Model 2
Score 0	Reference	Reference	Reference	Reference	Reference	Reference	Reference	Reference
Score 1	0.77 (0.42-1.41)	0.73 (0.4-1.35)	1.19 (1.13-1.25)	1.11 (1.06-1.17)	1.12 (1.05-1.19)	1.08 (1.01-1.15)	1.09(1.06-1.12)	1.04(1.02-1.07)
Score 2	1.81 (0.95-3.46)	1.57 (0.81-3.05)	1.37 (1.28-1.46)	1.19 (1.11-1.27)	1.14 (1.05-1.25)	1.07 (0.98-1.17)	1.16(1.11-1.2)	1.07(1.03-1.11)
Score 3	1.86 (0.74-4.68)	1.26 (0.48-3.31)	1.5 (1.37-1.67)	1.18 (1.07-1.3)	1.35 (1.2-1.51)	1.19 (1.07-1.34)	1.21(1.14-1.27)	1.06(1-1.11)
Score 4	2.66 (0.82-8.59)	1.48 (0.44-4.98)	1.89 (1.67-2.15)	1.34 (1.18-1.53)	1.4 (1.19-1.64)	1.17 (1.0-1.37)	1.38(1.28-1.48)	1.14(1.06-1.22)
Score ≥5	9.31 (4.17-20.79)	3.98 (1.69-9.39)	2.16 (1.88-2.48)	1.31 (1.14-1.51)	1.6 (1.36-1.89)	1.2 (1.02-1.41)	1.52(1.41-1.65)	1.16(1.07-1.26)
Multimorbidity defined by two or more diseases compared with no or one disease.								
Score ≥2	2.55 (1.62-4.02)	1.89 (1.17-3.05)	1.43 (1.36-1.51)	1.18 (1.12-1.24)	1.23 (1.15-1.31)	1.1 (1.03-1.18)	1.19(1.16-1.23)	1.07(1.04-1.1)

Abbreviations: OR=odds ratio. The ORs were derived from double entry and the variance covariance (vce) cluster method was used. Model 1 is a crude model. Model 2 is adjusted for sex, year of birth, county, and educational attainment.

For peer review only - http://bmjopen.bmj.com/site/about/guidelines.xhtml

Discussion

The strong association between multimorbidity and VTE is, to the best of our knowledge, a novel finding. The strong and increasing association with increasing multimorbidity score suggests that a possible causal association may exist between multimorbidity and VTE.[30] Our present study shows an association between multimorbidity and VTE with an adjusted OR of 3.16. The VTE risk increases with a higher multimorbidity score and is nine times higher in individuals with a multimorbidity score of five or more compared to individuals with no morbidity. Thus, multimorbidity is strongly associated with VTE. However, our data argue against a major genetic association between multimorbidity and VTE due to the mostly weak familial associations. However, there was a significant familial association, which correlated with the degree of relatedness suggesting a possible genetic contribution to the association between multimorbidity and VTE. A high multimorbidity score in a patient confers similar VTE risk as strong genetic or acquired risk factors.[1,2,6,10-12] Based on these findings it is not unlikely that multimorbidity will turn out to be included in risk stratification for antithrombotic treatment during hospitalization, surgery and immobilization. Multimorbidity might also be of value in diagnostic algorithms of VTE and for follow-up after VTE.

The impact of multimorbidity on VTE is stronger in males compared to females and with a higher relationship among males. This knowledge could be useful in the clinical setting when considering diagnostics. On the other hand, females have increased multimorbidity and live longer which may give a double burden for the female population in the context risk of VTE.

The risk of multimorbidity is increasing with age and more individuals worldwide are multimorbid.[16] Our study population is relatively young and confirms previous studies that

For peer review only - http://bmjopen.bmj.com/site/about/guidelines.xhtml

multimorbidity may also affect young persons.[15] However, there was a strong association with multimorbidity in all age groups suggesting that multimorbidity might also be of importance in older age groups not studied here; this needs to be confirmed in further studies. It is possible that multimorbidity is an important contributor to the high incidence of VTE in society due to its high prevalence. Common risk factors, both genetic and acquired, are often seen as individual risk factors in specific settings, like surgery, pregnancy, puerperium, oral contraceptives, surgery and trauma as well as for specific diseases, such as cancer. The present study suggests that we might count the total burden of disease into a multimorbidity score to get a better risk estimate of VTE.

Since there is no clear definition of which diagnoses should be used in the field of multimorbidity the ones that are included are sometimes random.[14-18] In this study we used a modified score validated by Barnett et al. 2012.[14] The NCDs that were used in this study are all common. They all have an impact on healthcare, and most are considerably stable during an individual's life and can coexist and create a significant health burden. It will be important to investigate whether a certain combination of diagnoses and multimorbidity has an especially high risk of VTE and further studies are needed to investigate this matter. However, the association between multimorbidity and VTE suggests that not only specific diseases are important but also the total burden of disease.

Strengths and limitations

A limitation of the study is that we did not determine any temporal association between multimorbidity and VTE. The study period was chosen to minimize the risk of missed data and to increase the coverage of individuals. Furthermore, having more comprehensive ICD-10

For peer review only - http://bmjopen.bmj.com/site/about/guidelines.xhtml

codes decreases the risk of inaccurate coding from the registers. A strength of the study is the high coverage and high validity of the registers and the large number of unique individuals.[20-25] A limitation is that only Swedish-born individuals born by Swedish parents were included. It therefore remains for a future study to show if the results may be generalized to other populations. Another limitation is that the data did not include biological and environmental risk factors such as smoking, alcohol consumption, height, weight, blood pressure and exercise habits. To make up for the absence of such data we adjusted for educational level, which is related to lifestyle factors.[29] Also, the included patients were relatively young with an age-range of 0-68 and a mean of 32 at the end of the study and generalization of the elderly is uncertain, even though within this age span between 0-68 years there was no age effect on the association. Hence there is a need for further studies in the future.

Conclusion

We have shown that an association exists between multimorbidity and venous thromboembolism. Multimorbidity might be an important factor contributing to the high incidence of VTE in society.

Further studies need to be done to map which of the diseases in the multimorbid patient contributes most to the increased risk of VTE and to develop tools to refine the diagnostics enhancement and to distinguish which patients may have use of prophylactic treatment.

For peer review only

For peer review only - http://bmjopen.bmj.com/site/about/guidelines.xhtml

Patient and Public Involvement statement

No patients or members of the public were involved.

Sources of funding

ALF-funding from Region Skåne (N/A) and the Swedish Research Council (2020-01824).

Acknowledgments

We thank Patrick Reilly, science editor at Center for Primary Health Care Research, Department of Clinical Science, Lund University and Region Skåne, Malmö, Sweden, for language review.

Author contributions

All authors contributed to the conception and design of the study; JS and KS contributed to the acquisition of data; all authors contributed to the analysis and interpretation of data; JA drafted the manuscript; and all authors revised it critically and approved the final version. All authors had full access to all the data (including statistical reports and tables) and take responsibility for the integrity of the data and the accuracy of their analysis.

Disclosure of Conflict of Interest

The authors have nothing to disclose.

Data sharing statement

Data for this original research article will be shared with external scientists in accordance with Swedish law and the agreements that exist between us and the Swedish Government agencies.

For peer review only - http://bmjopen.bmj.com/site/about/guidelines.xhtml

For peer review only

References

1. White RH. The epidemiology of venous thromboembolism. Circulation. 2003;107(23 Suppl 1):I4-8.
2. Heit JA. Epidemiology of venous thromboembolism. Nat Rev Cardiol. 2015;12:464-74.
3. Hansson PO, Welin L, Tibblin G, Eriksson H. Deep vein thrombosis and pulmonary embolism in the general population. 'The Study of Men Born in 1913'. Arch Intern Med. 1997;157(15):1665-70.
4. Manderstedt E, Halldén C, Lind-Halldén C, Elf J, Svensson PJ, Engström G, Melander O, Baras A, Lotta LA, Zöller B; Regeneron Genetics Center. Thrombomodulin (THBD) gene variants and thrombotic risk in a population-based cohort study. J Thromb Haemost. 2022;20(4):929-935.
5. Naess IA, Christiansen SC, Romundstad P, Cannegieter SC, Rosendaal FR, Hammerstrøm J. Incidence and mortality of venous thrombosis: a population-based study. J Thromb Haemost. 2007;5(4):692-9.
6. Rosendaal FR. Venous thrombosis: a multicausal disease. Lancet. 1999;353(9159):1167-73.
7. Brandjes DP, Büller HR, Heijboer H, Huisman MV, de Rijk M, Jagt H, ten Cate JW. Randomised trial of effect of compression stockings in patients with symptomatic proximal-vein thrombosis. Lancet. 1997;349(9054):759-62.
8. Anderson FA Jr, Wheeler HB, Goldberg RJ, Hosmer DW, Patwardhan NA, Jovanovic B, Forcier A, Dalen JE. A population-based perspective of the hospital incidence and case-fatality rates of deep vein thrombosis and pulmonary embolism. The Worcester DVT Study. Arch Intern Med. 1991;151(5):933-8.
9. Zöller B, Pirouzifard M, Sundquist J, Sundquist K. Association of Short-Term Mortality of Venous Thromboembolism with Family History of Venous Thromboembolism and Charlson Comorbidity Index. Thromb Haemost. 2019;119(1):48-55.
10. Zöller B, Svensson PJ, Dahlbäck B, Lind-Hallden C, Hallden C, Elf J. Genetic risk factors for venous thromboembolism. Expert Rev Hematol. 2020;13(9):971-981.
11. Manderstedt E, Manderstedt E, Lind-Halldén C, Halldén C, Elf J, Svensson PJ, Dahlbäck B, Engström G, Melander O, Baras A, Lotta LA, Zöller B; Regeneron Genetics Center 4 [Link]. Classic Thrombophilias and Thrombotic Risk Among Middle-Aged and Older Adults: A Population-Based Cohort Study. J Am Heart Assoc. 2022;11(4):e023018.
12. Lijfering WM, Rosendaal FR, Cannegieter SC. Risk factors for venous thrombosis - current understanding from an epidemiological point of view. Br J Haematol. 2010;149(6):824-33.
13. Zöller B, Li X, Sundquist J, Sundquist K. Risk of pulmonary embolism in patients with autoimmune disorders: a nationwide follow-up study from Sweden. Lancet. 2012;379(9812):244-9.
14. Barnett K, Mercer SW, Norbury M, Watt G, Wyke S, Guthrie B. Epidemiology of multimorbidity and implications for health care, research, and medical education: a cross-sectional study. Lancet. 2012;380(9836):37-43.

For peer review only - http://bmjopen.bmj.com/site/about/guidelines.xhtml

15. Troelstra SA, Straker L, Harris M, Brown S, van der Beek AJ, Coenen P. Multimorbidity is common among young workers and related to increased work absenteeism and presenteeism: results from the population-based Raine Study cohort. Scand J Work Environ Health. 2020;46(2):218-227.
16. Johnston MC, Crilly M, Black C, Prescott GJ, Mercer SW. Defining and measuring multimorbidity: a systematic review of systematic reviews. Eur J Public Health. 2019 Feb 1;29(1):182-189.
17. Pearson-Stuttard J, Ezzati M, Gregg EW. Multimorbidity-a defining challenge for health systems. Lancet Public Health. 2019;4(12):e599-e600.
18. Wallace E, Salisbury C, Guthrie B, Lewis C, Fahey T, Smith SM. Managing patients with multimorbidity in primary care. BMJ. 2015;350:h176.
19. Risch N. The genetic epidemiology of cancer: interpreting family and twin studies and their implications for molecular genetic approaches. Cancer Epidemiol Biomarkers Prev. 2001;10:733-41
20. Rosen M, Hakulinen T. Use of disease registers. In: Ahrens W, Pigeot I, eds. Handbook of Epidemiology. Springer-Verlag; 2005:231–252.
21. Ludvigsson JF, Otterblad-Olausson P, Pettersson BU, Ekbom A. The Swedish personal identity number: possibilities and pitfalls in healthcare and medical research. Eur J Epidemiol. 2009;24(11):659-67.
22. Ludvigsson JF, Andersson E, Ekbom A, Feychting M, Kim JL, Reuterwall C, Heurgren M, Olausson PO. External review and validation of the Swedish national inpatient register. BMC Public Health. 2011;11:450.
23. Ludvigsson JF, Almqvist C, Bonamy AK, Ljung R, Michaëlsson K, Neovius M, Stephansson O, Ye W. Registers of the Swedish total population and their use in medical research. Eur J Epidemiol. 2016;31(2):125-36.
24. Ekbom A. The Swedish Multi-generation Register. Methods Mol Biol. 2011;675:215-20.
25. Zöller B. Nationwide family studies of cardiovascular diseases—clinical and genetic implications of family history. EMJ Cardiol. 2013;1:102–113.
26. Kohler HP, Rodgers JL. DF-analyses of heritability with double-entry twin data: asymptotic standard errors and efficient estimation. Behav Genet. 2001 Mar;31(2):179-91.
27. Rosengren A, Fredén M, Hansson PO, Wilhelmsen L, Wedel H, Eriksson H. Psychosocial factors and venous thromboembolism: a long-term follow-up study of Swedish men. J Thromb Haemost. 2008;6(4):558-64.
28. McLoughlin G, Palmer JA, Rijsdijk F, Makeig S. Genetic overlap between evoked frontocentral theta-band phase variability, reaction time variability, and attention-deficit/hyperactivity disorder symptoms in a twin study. Biol Psychiatry. 2014; 75: 238-47.
29. Winkleby MA, Jatulis DE, Frank E, Fortmann SP. Socioeconomic status and health: how education, income, and occupation contribute to risk factors for cardiovascular disease. Am J Public Health. 1992;82(6):816-20.
30. Hill AB. The environment and disease: association or causation? Proc R Soc Med. 1965;58(5):295-300.

For peer review only - http://bmjopen.bmj.com/site/about/guidelines.xhtml

Supplementary Appendix: Authors: Ahrén J, Pirouzifard M, Holmquist B, Sundquist J, Halling A, Sundquist K, Zöller B

Table of content pages 1-9 **Pages**

1. Supplementary Table S1 2-3
2. Supplementary Table S2 4
3. Supplementary Table S3 5
4. Supplementary Table S4 6
5. Supplementary Table S5 7
6. Supplementary Table S6 8
7. Supplementary Table S7 9

For peer review only

Supplementary Table S1. Diseases with ICD codes included in the multimorbidity score modified and adapted to Swedish ICD-10 codes after Barnett K et al (2012)*. Infectious diseases, i.e. viral hepatitis, were excluded and six common non-communicable diseases (NCDs) were added: dermatitis or eczema, gout, arthrosis, osteoporosis, obesity, and pancreatitis. One point for each disease. The theoretical range of the score is 0-45 points.

	Included disorders in the multimorbidity score	ICD-10**
1	Hypertension	I10-I15
2	Affective disorders	F30-F39
3	Painful back condition	M50-54
4	Asthma	J45-J46
5	CHD	I20-I25
6	Ulcer disease	K221, K25-K28
7	Diabetes	E10-E14
8	Thyroid disorders	E00-E07
9	Connective tissue disease	M05, M06, M08, M09, M30-M36, D86
10	Hearing loss (or impaired)	H90-H91
11	Chronic obstructive pulmonary disease (COPD)	J41-J44
12	Anxiety	F40-F48
13	Irritable bowel syndrome (IBS)	K58
14	Cancer	C00-C99
15	Alcohol use disorders	F10
16	Psychoactive substance misuse	F11-F14, F16, F18, F19
17	Constipation	K590
18	Cerebrovascular disease	I60-I69
19	Renal disease	N00-N19, Q61
20	Diverticular disease of intestine	K57
21	Atrial fibrillation	I48
22	Atherosclerosis	I70-I74, I77
23	Heart failure	I50, I110, I130, I132
24	Prostate disease	N40-N42
25	Glaucoma	H40, H42

For peer review only - http://bmjopen.bmj.com/site/about/guidelines.xhtml

For peer review only

26	Epilepsy	G40, G41
27	Dementia	F00-F03
28	Schizophrenia disorders	F20, F21
29	Psoriasis	L40
30	Dermatitis or Eczema	L20-L30
31	Inflammatory bowel disease (IBD)	K50, K51
32	Migraine	G43, G440, G441
33	Blindness & low vision	H53-H54
34	Chronic sinusitis	J32
35	Learning disability	F81
36	Anorexia or bulimia	F50
37	Bronchiectasis	J47
38	Parkinson's disease	G20, G21, G22
39	Multiple sclerosis	G35
40	Liver disease	K70-K77
41	Gout	M10
42	Arthrosis	M15-M19
43	Osteoporosis	M80-M82
44	Obesity	E65, E66
45	Pancreas diseases	K85, K86

*Barnett K, Mercer SW, Norbury M, Watt G, Wyke S, Guthrie B. Epidemiology of multimorbidity and implications for health care, research, and medical education: a cross-sectional study. Lancet 2012; 380: 37-43.

**ICD=International classification of disease.

Supplementary Table S2. Odds ratio for VTE according to multimorbidity score (0 to ≥5) in males. Odds Ratios (ORs) with 95 % confidence interval (CI) for multimorbidity scores. Reference with no diseases (score=0).

For peer review only

	Male (n=1381453)			
	OR (95% CI)			
VTE score	No VTE	VTE	Model 1	Model 2
Score 0	882367	2509	1[Reference]	1[Reference]
Score 1	307866	1900	2.17 (2.04-2.30)	2.03 (1.91-2.26)
Score 2	111533	1189	3.75 (3.5-4.02)	3.20 (2.99-3.44)
Score 3	42063	763	6.38 (5.88-6.92)	4.73 (4.35-5.14)
Score 4	17441	483	9.74 (8.83-10.75)	6.3(5.69-6.96)
Score ≥5	12695	644	17.84 (16.33-19.49)	9.38 (8.55-10.28)

Abbreviations: OR, odds ratio. Model 1 is a crude model (univariate). Model 2 is an adjusted model (multivariate), with adjustments for year of birth, county, and educational attainment.

For peer review only

Supplementary Table S3. Odds ratio for VTE according to multimorbidity score (0 to ≥5) in females. Odds Ratios (ORs) with 95 % confidence interval (CI) for multimorbidity scores. Reference with no diseases (score=0).

	Female (n=1312989)			
	OR (95% CI)			
VTE score	No VTE	VTE	Model 1	Model 2
Score 0	728838	2499	1[Reference]	1[Reference]
Score 1	325513	2208	1.98 (1.88-2.10)	1.84 (1.73-1.94)
Score 2	140025	1463	3.05 (2.86-3.25)	2.67 (2.5-2.85)
Score 3	60906	902	4.32 (4.0-4.66)	3.53 (3.26-3.81)
Score 4	27081	580	6.25 (5.70-6.84)	4.76 (4.34-5.22)
Score ≥5	22015	959	12.70 (11.78-13.70)	8.58 (7.94-9.27)

Abbreviations: OR, odds ratio. Model 1 is a crude model (univariate). Model 2 is an adjusted model (multivariate), with adjustments for year of birth, county, and educational attainment.

Supplementary Table S4. Odds ratio for VTE according to multimorbidity score (0 to ≥5) for individuals born 1947-1972. Odds Ratios (ORs) with 95 % confidence interval (CI) for multimorbidity scores. Reference with no diseases (score=0).

	All (n=641211)			
	OR (95% CI)			
VTE score	NO VTE	VTE	Model 1	Model 2
Score 0	340353	2331	1[Reference]	1[Reference]
Score 1	156444	2062	1.92 (1.78-2.00)	1.89 (1.78-2.00)
Score 2	70005	1437	3 (2.81-3.2)	2.89 (2.7-3.08)
Score 3	33176	957	4.22 (3.9-4.54)	4 (3.7-4.31)
Score 4	16542	624	5.51 (5.04-6.02)	5.13 (4.68-5.62)
Scor	16266	1014	9.1 (8.44-9.81)	8.3 (7.68-8.97)

For peer review only - http://bmjopen.bmj.com/site/about/guidelines.xhtml

e ≥5				

Abbreviations: OR, odds ratio. Model 1 is a crude model (univariate). Model 2 is an adjusted model (multivariate), with adjustments for sex, year of birth, county, and educational attainment.

For peer review only

Supplementary Table S5. Odds ratio for VTE according to multimorbidity score (0 to ≥5) for individuals born 1972-1983. Odds Ratios (ORs) with 95 % confidence interval (CI) for multimorbidity scores. Reference with no diseases (score=0).

	All (n=682582)			
	OR (95% CI)			
VTE score	NO VTE	VTE	Model 1	Model 2
Score 0	415987	1638	1[Reference]	1[Reference]
Score 1	155822	1277	2.08 (1.93-2.24)	2.01 (1.87-2.16)
Score 2	60801	725	3.02 (2.77-3.31)	2.85 (2.61-3.11)
Score 3	25237	468	4.71 (4.25-5.22)	4.33 (3.9-4.81)
Score 4	11184	280	6.36 (5.59-7.23)	5.74 (5.04-6.54)
Score ≥5	8779	384	11.11 (9.92-12.44)	9.7 (8.64-10.89)

Abbreviations: OR, odds ratio. Model 1 is a crude model (univariate). Model 2 is an adjusted model (multivariate), with adjustments for sex, year of birth, county, and educational attainment.

Supplementary Table S6. Odds ratio for VTE according to multimorbidity score (0 to ≥5) for individuals born 1983-1993. Odds Ratios (ORs) with 95 % confidence interval (CI) for multimorbidity scores. Reference with no diseases (score=0).

	All (n=691267)			
	OR (95% CI)			
VTE score	NO VTE	VTE	Model 1	Model 2
Score 0	427804	845	1[Reference]	1[Reference]
Score 1	156178	624	2.02 (1.82-2.24)	1.94 (1.75-2.15)
Score 2	61537	399	3.28 (2.91-3.7)	3.04 (2.69-3.43)
Score 3	25768	204	4.01 (3.44-4.67)	3.59 (3.07-4.2)
Score 4	10734	137	6.46 (5.39-7.75)	5.63 (4.68-6.78)
Score ≥5	6858	179	13.21 (11.23-15.55)	10.91 (9.21-12.91)

For peer review only - http://bmjopen.bmj.com/site/about/guidelines.xhtml

Abbreviations: OR, odds ratio. Model 1 is a crude model (univariate). Model 2 is an adjusted model (multivariate), with adjustments for sex, year of birth, county, and educational attainment.

Supplementary Table S7. Odds ratio for VTE according to multimorbidity score (0 to ≥5) for individuals born 1993-2005. Odds Ratios (ORs) with 95 % confidence interval (CI) for multimorbidity scores. Reference with no diseases (score=0).

	All (n=679382)			
	OR (95% CI)			
VTE score	NO VTE	VTE	Model 1	Model 2
Score 0	427061	194	1[Reference]	1[Reference]
Score 1	164935	145	1.94 (1.56-2.4)	1.94 (1.56-2.41)
Score 2	59215	91	3.38 (2.64-4.34)	3.16 (2.46-4.06)
Score 3	18788	36	4.22 (2.95-6.02)	3.49 (2.44-5)
Score 4	6062	22	7.99 (5.14-12.43)	5.92 (3.8-9.24)
Score ≥5	2807	26	20.4 (13.52-30.76)	13.46 (8.87-20.42)

Abbreviations: OR, odds ratio. Model 1 is a crude model (univariate). Model 2 is an adjusted model (multivariate), with adjustments for sex, year of birth, county, and educational attainment.

For peer review only - http://bmjopen.bmj.com/site/about/guidelines.xhtml

STROBE 2007 (v4) Statement—Checklist of items that should be included in reports of *cross-sectional studies*

Section/Topic	Item #	Recommendation	Reported on page #
**Title and abstract**	1	(*a*) Indicate the study's design with a commonly used term in the title or the abstract	3
		(*b*) Provide in the abstract an informative and balanced summary of what was done and what was found	3
**Introduction**			
Background/rationale	2	Explain the scientific background and rationale for the investigation being reported	6-7
Objectives	3	State specific objectives, including any prespecified hypotheses	7
**Methods**			
Study design	4	Present key elements of study design early in the paper	8-9
Setting	5	Describe the setting, locations, and relevant dates, including periods of recruitment, exposure, follow-up, and data collection	8-9
Participants	6	(*a*) Give the eligibility criteria, and the sources and methods of selection of participants	8-9
Variables	7	Clearly define all outcomes, exposures, predictors, potential confounders, and effect modifiers. Give diagnostic criteria, if applicable	9-10
Data sources/ measurement	8*	For each variable of interest, give sources of data and details of methods of assessment (measurement). Describe comparability of assessment methods if there is more than one group	9-10
Bias	9	Describe any efforts to address potential sources of bias	*10-11*
Study size	10	Explain how the study size was arrived at	8-12
Quantitative variables	11	Explain how quantitative variables were handled in the analyses. If applicable, describe which groupings were chosen and why	8-11
Statistical methods	12	(*a*) Describe all statistical methods, including those used to control for confounding	10-11
		(*b*) Describe any methods used to examine subgroups and interactions	10-11
		(*c*) Explain how missing data were addressed	10-11
		(*d*) If applicable, describe analytical methods taking account of sampling strategy	10-11
		(*e*) Describe any sensitivity analyses	10-11
**Results**			

For peer review only

Participants	13*	(a) Report numbers of individuals at each stage of study—eg numbers potentially eligible, examined for eligibility, confirmed eligible, included in the study, completing follow-up, and analysed	12-17
		(b) Give reasons for non-participation at each stage	NA -nationwide study
		(c) Consider use of a flow diagram	Not used
Descriptive data	14*	(a) Give characteristics of study participants (eg demographic, clinical, social) and information on exposures and potential confounders	12-17
		(b) Indicate number of participants with missing data for each variable of interest	NA -nationwide study
Outcome data	15*	Report numbers of outcome events or summary measures	12-17
Main results	16	(*a*) Give unadjusted estimates and, if applicable, confounder-adjusted estimates and their precision (eg, 95% confidence interval). Make clear which confounders were adjusted for and why they were included	12-17
		(*b*) Report category boundaries when continuous variables were categorized	12-17
		(*c*) If relevant, consider translating estimates of relative risk into absolute risk for a meaningful time period	12-17
Other analyses	17	Report other analyses done—eg analyses of subgroups and interactions, and sensitivity analyses	12-17
**Discussion**			
Key results	18	Summarise key results with reference to study objectives	18-20
Limitations	19	Discuss limitations of the study, taking into account sources of potential bias or imprecision. Discuss both direction and magnitude of any potential bias	18-20
Interpretation	20	Give a cautious overall interpretation of results considering objectives, limitations, multiplicity of analyses, results from similar studies, and other relevant evidence	18-20
Generalisability	21	Discuss the generalisability (external validity) of the study results	18-20
**Other information**			
Funding	22	Give the source of funding and the role of the funders for the present study and, if applicable, for the original study on which the present article is based	21

*Give information separately for cases and controls in case-control studies and, if applicable, for exposed and unexposed groups in cohort and cross-sectional studies.

Note: An Explanation and Elaboration article discusses each checklist item and gives methodological background and published examples of transparent reporting. The STROBE checklist is best used in conjunction with this article (freely available on the Web sites of PLoS Medicine at http://www.plosmedicine.org/, Annals of Internal Medicine at http://www.annals.org/, and Epidemiology at http://www.epidem.com/). Information on the STROBE Initiative is available at www.strobe-statement.org.

For peer review only

For peer review only

BMJ Open

A hypothesis - generating Swedish extended national cross-sectional family study of multimorbidity severity and venous thromboembolism

Journal:	*BMJ Open*
Manuscript ID	bmjopen-2023-072934.R1
Article Type:	Original research
Date Submitted by the Author:	19-Apr-2023
Complete List of Authors:	Ahrén, Jonatan; Lund University, Center for Primary Health Care Research<br>Pirouzifard, Mirnabi; Lund University, Center for Primary Health Care Research<br>Holmquist, Björn; Lund University Department of Statistics, Department of Statistics<br>Sundquist, Jan; Lund University, Center for Primary Health Care Research<br>Halling, Anders; Lunds Universitet, Center for Primary Health Care Research<br>Sundquist, Kristina; Lund University, Center for Primary Health Care Research<br>Zöller, Bengt; Lund University, Center for Primary Health Care Research
**Primary Subject Heading**:	Cardiovascular medicine
Secondary Subject Heading:	Epidemiology, General practice / Family practice
Keywords:	Thromboembolism < CARDIOLOGY, EPIDEMIOLOGY, GENERAL MEDICINE (see Internal Medicine), Vascular medicine < INTERNAL MEDICINE, Primary Health Care

SCHOLARONE™
Manuscripts

I, the Submitting Author has the right to grant and does grant on behalf of all authors of the Work (as defined in the below author licence), an exclusive licence and/or a non-exclusive licence for contributions from authors who are: i) UK Crown employees; ii) where BMJ has agreed a CC-BY licence shall apply, and/or iii) in accordance with the terms applicable for US Federal Government officers or employees acting as part of their official duties; on a worldwide, perpetual, irrevocable, royalty-free basis to BMJ Publishing Group Ltd ("BMJ") its licensees and where the relevant Journal is co-owned by BMJ to the co-owners of the Journal, to publish the Work in this journal and any other BMJ products and to exploit all rights, as set out in our licence.

The Submitting Author accepts and understands that any supply made under these terms is made by BMJ to the Submitting Author unless you are acting as an employee on behalf of your employer or a postgraduate student of an affiliated institution which is paying any applicable article publishing charge ("APC") for Open Access articles. Where the Submitting Author wishes to make the Work available on an Open Access basis (and intends to pay the relevant APC), the terms of reuse of such Open Access shall be governed by a Creative Commons licence – details of these licences and which Creative Commons licence will apply to this Work are set out in our licence referred to above.

Other than as permitted in any relevant BMJ Author's Self Archiving Policies, I confirm this Work has not been accepted for publication elsewhere, is not being considered for publication elsewhere and does not duplicate material already published. I confirm all authors consent to publication of this Work and authorise the granting of this licence.

For peer review only

A hypothesis - generating Swedish extended national cross-sectional family study of multimorbidity severity and venous thromboembolism

[1]Jonatan Ahrén MD, [1]MirNabi Pirouzifard, PhD; [2]Björn Holmquist, PhD; [1]Jan Sundquist, MD, PhD; [1]Anders Halling PhD, MD; [1]Kristina Sundquist, MD, PhD, [1]Bengt Zöller MD, PhD.

[1]Center for Primary Health Care Research, Lund University/Region Skåne, Malmö, Sweden.

[2]Department of Statistics Lund University Lund Sweden

For peer review only

Running Title: Multimorbidity and venous thromboembolism

Correspondence:

Dr Jonatan Ahrén

Center for Primary Health Care Research

CRC, Building 28, Floor 11

Jan Waldenströms gata 35

Skåne University Hospital

S-205 02 Malmö, Sweden

Tel: +46 735127050; Fax: +46 40 391370

jonatan.ahren@med.lu.se

Word count: 3830 including tables **Abstract word count:** 300

Keywords: epidemiology, genetics medical, medicine, multimorbidity, public health, venous thromboembolism

For peer review only - http://bmjopen.bmj.com/site/about/guidelines.xhtml

Abstract

Objectives: Venous thromboembolism (VTE) is a common worldwide disease. The burden of multimorbidity, i.e. two or more chronic diseases, has increased. Whether multimorbidity is associated with VTE risk remains to be studied. Our aim was to determine any association between multimorbidity and VTE and any possible shared familial susceptibility.

Design: A nationwide extended cross-sectional hypothesis - generating family study between 1997-2015.

Setting: The Swedish Multi-Generation Register, the National Patient Register, the Total Population Register and the Swedish cause of death register were linked.

Participants: 2,694,442 unique individuals were analyzed for VTE and multimorbidity.

Main Outcomes and Measures: Multimorbidity was determined by a counting method using 45 non-communicable diseases (NCD). Multimorbidity was defined by the occurrence of ≥2 diseases. A multimorbidity score was constructed defined by, 0, 1, 2, 3, 4 or 5 or more diseases.

Results: Sixteen percent (n=440742) of the study population was multimorbid. Of the multimorbid patients 58 % were females. There was an association between multimorbidity and VTE. The adjusted odds ratio (OR) for VTE in individuals with multimorbidity (2≥diagnoses) was 3.16 (95%CI 3.06-3.27) compared to individuals without multimorbidity. There was an association between number of diseases and VTE. The adjusted OR was 1.94 (95%CI 1.86-2.02) for one disease, 2.93 (95%CI 2.80-3.08) for two diseases, 4.07 (95%CI 3.85-4.31) for three diseases, 5.46 (95%CI 5.10-5.85) for four diseases, and 9.08 (95%CI 8.56-9.64) for 5≥ diseases. The association between multimorbidity and VTE was stronger in males OR 3.45 (3.29-3.62) than in females OR 2.91 (2.77-3.04). There were significant but mostly weak familial associations between multimorbidity in relatives and VTE.

For peer review only

Conclusions: Increasing multimorbidity exhibits a strong and increasing association with VTE. Familial associations suggest a weak shared familial susceptibility. The association between multimorbidity and VTE suggests that future cohort studies where multimorbidity is used to predict VTE might be worthwhile.

For peer review only

For peer review only - http://bmjopen.bmj.com/site/about/guidelines.xhtml

Strength and limitations of this study

- High coverage and high validity of the registers and the large number of unique individuals.

- Only Swedish-born individuals born by Swedish parents were included. It therefore remains for a future study to show if the results may be generalized to other populations.

- The data did not include biological and environmental risk factors, to make up for the absence of such data we adjusted for educational level, which is related to lifestyle factors.

-Due to the cross-sectional design a temporal and possible causal association remains to be determined.

For peer review only

Introduction

Venous thromboembolism (VTE) is the third most common cardiovascular disease with an incidence of around 1-3 per 1000 individuals.[1-4] The incidence rate increases with age. VTE is rare among children with an incidence of approximately 1 per 100000 and common among elderly people >70 years and above with an incidence of around 1% per year.[5] The lifetime risk of VTE could be up to 10% in certain countries, for instance in Sweden.[3,4] VTE usually manifests as deep venous thrombosis (DVT) of the lower extremities or as pulmonary embolism (PE).[6] The consequences of VTE range from post-thrombotic syndrome, which affects approximately 20% of patients with DVT, to acute death in 1-2% of PE patients.[7,8] The 90-day mortality after VTE is around 5%.[9]

Risk factors for VTE are either acquired or genetic but they often interact.[8] Among the genetic risk factors, the most prominent are the classical thrombophilia, i.e. factor V Leiden (rs6025), the prothrombin G20210A mutation (rs1799963), deficiencies of antithrombin, protein C and protein S.[10,11] Among the acquired risk factors, age is the most significant with a steeply increased risk with advanced age.[5,6] Other acquired risk factors are for instance immobilization, trauma and surgery, malignancy, pregnancy and puerperium, oral contraception, and autoimmune disorders.[1,2,6,12,13]

Risk factors for VTE may work alone or in concert.[6,12] Multimorbidity, i.e. the presence of two or more diseases, increases with age.[14] As patients get older and the field of medicine advances the patients more often present with multiple diseases - multimorbidity.[14] However, multimorbidity also occurs among younger persons.[15] Genetic inheritance has been implicated in multimorbidity.[16] Due to this there is a need to investigate multimorbidity as a risk factor

For peer review only - http://bmjopen.bmj.com/site/about/guidelines.xhtml

for additional illnesses, such as VTE. With the future demographic change with an increasingly elderly population with multimorbidity, there may be a need to adopt a multi-disease framework for clinical diagnostics and treatment. Despite the need to characterize multimorbidity there is no established definition of which morbidities should be included to define multimorbidity.[17] Multimorbidity has often been defined as two or more chronic non-communicable diseases (NCD).[17,18] Barnett et al used 40 diseases in a Scottish cross-sectional study and defined multimorbidity as the presence of two or more diseases where the prevalence of multimorbidity was 23.2% and was present in most people 65 years of age and older.[14] Although the frequency of multimorbidity increases with age, the burden of multimorbidity may be more common in those aged 65 years or younger, and this is especially common in socially deprived neighborhoods.[19] Several NCDs, but not all, included in the multimorbidity study by Barnett et al[14] have been associated with VTE.[20-39] For some associations between NCDs and VTE the associations are bidirectional,[36-38] e.g atherosclerotic disease and atrial fibrillation, but for most NCDs the association with VTE is unidirectional.[20-35] However, to the best of our knowledge, the association between multimorbidity and its severity (i.e. number of NCDs) and VTE has not been studied.

We aimed to determine whether there is an association between multimorbidity and its severity (i.e. number of NCDS) and VTE. We used large Swedish national registers to study the association between multimorbidity and VTE. A modified list of 45 NCDs diagnoses used by Barnett et al was used to define multimorbidity as the presence of two or more of 45 NCDs.[14] To determine a possible association between multimorbidity and VTE we also examined the association between the number of diseases and VTE. We also investigated whether a familial association of multimorbidity and VTE exists. Familial associations of a disease may be due to shared environmental exposure(s), shared genetic susceptibility, or a

For peer review only - http://bmjopen.bmj.com/site/about/guidelines.xhtml

combination of the two [40] but familial association of a disease is a necessary, though insufficient, condition to infer the importance of genetic susceptibility.[40]

Material and methods

Registers

The following registers were used in the study: The Swedish Multi-Generation Register contains data of familial relationships; the National Patient Register (NPR) contains hospital discharge diagnoses between 1964-2015 with nationwide coverage from 1987 and from 2001-2015 outpatient hospital diagnoses; the Total Population Register with data on death date, education, marital status, migration; and the Swedish cause of death register.[41-46] We used the unique Swedish personal identity number (PIN) to link the databases. The PIN was, however, replaced with a serial number to ensure confidentiality by Statistics Sweden. The validity of Swedish registers has been investigated previously and they have a high degree of coverage and validity.[41-46] Statistics Sweden and the National Board of Health and Welfare provided the register data to us.

Study design

A nationwide extended cross-sectional study between 1997-2015. This period was chosen to increase the coverage of the registers and to use only the latest versions of the international classification of disease (ICD) 10 codes. To minimize the risk of an incorrect PIN only Swedish-born individuals were included.[42] To investigate any possible familial aggregation we included pairs of full siblings born between 1948 and 2005 by Swedish parents that could further be linked to their Swedish-born half-siblings, and cousins to minimize this source of

For peer review only - http://bmjopen.bmj.com/site/about/guidelines.xhtml

For peer review only

error. Parents in the study were born between 1932 and 1985 in Sweden. The Swedish National Multi-Generation Register was used to link relatives. We excluded the families with members who died or emigrated before 1997 or emigrated before the age of 17. Both biological parents were obligatorily known, i.e. pedigrees with two parents and two children. Full siblings were thereafter linked with related families and with the full siblings' half-siblings and cousins. Full siblings will hereafter be referred to as “siblings”. One dataset with all individuals entered only once was created. Four datasets for familial relations were created: twins, siblings, half-siblings, and cousins. In the four familial datasets, all relative pairs were double-entered, i.e. all sibling pairs, all twins’ pairs, all half-sibling pairs, and all cousin pairs, as described previously.[47] The double entry approach is a common procedure in genetics.[47,48] Zygosity was assessed only from sex, i.e. twins with different sex were considered to be DZ twins. We had no access to zygosity data. We allowed the same person to be included in more than one family relationship.

Definition of venous thromboembolism

Cases of VTE (i.e., affection status) were classified according to the World Health Organization International Classification of Diseases, Tenth Revision (ICD-10). VTE was defined using the hospital discharge register, main and supplementary diagnoses (between 1997 and 2015) by the following ICD codes: ICD-10: I80 (not I80.0) and I26.[11] It was also defined in the Outpatient Care Register (between 2001 and 2015) by the same ICD-10 codes as above and the cause of death register. Thus, VTE included pulmonary embolism and deep vein thrombosis of the lower extremities. The diagnosis of VTE in the NPR has been shown to have an accuracy of 95%, whereas the overall validity of the NPR is 87%.[43,48] A quality control of the NPR register was performed for 118 patients with VTE in the Malmö diet and cancer study cohort. In 106 (90%) of 118 cases the diagnosis of VTE was correct.[4]

For peer review only - http://bmjopen.bmj.com/site/about/guidelines.xhtml

Multimorbidity score

Since there is no clear definition of which NCDs should be included, the NCDs used in Barnett et al. were used and modified to adapt to the Swedish ICD-10 codes (Supplementary Table S1). Further modifications were made to the list and infectious diseases were excluded. Five other clinically important and common NCDs contributing to disease burden were added instead: pancreatitis, obesity, gout, arthrosis, and osteoporosis. Furthermore, psoriasis and dermatitis or eczema were counted separately. These modifications were made to increase the number of NCDs to get a more comprehensive list. Thus, a total of 45 diagnoses were included and an individual with two or more NCDs was considered to have multimorbidity. Each diagnosis in an individual during the study period was awarded one point per disease and was cumulatively counted. An individual could theoretically have 45 points. In the study, no individual obtained more than 20 points.

Statistical analysis

Logistic regression was used to investigate the association between VTE and multimorbidity. Multimorbidity was defined as two or more diseases. Multimorbidity was also investigated as a cumulative score in order to determine any possible increasing association. Results are presented as odds ratios (ORs) with 95% confidence intervals (95%CI). Adjustments were made for year of birth, sex, region at birth and level of education. Stratified analysis was performed according to sex and birth year quartiles. The familial associations were determined using the double-entry approach.[47] The ambiguity in unselected samples as to which twin's, sibling's, half-sibling's or cousin's trait should be used as the dependent, and which as the independent variable, could be resolved by using double-entry.[47] Each twin, sibling, half-sibling, or cousin is entered twice in the data, and each member of a twin, sibling,

For peer review only - http://bmjopen.bmj.com/site/about/guidelines.xhtml

half-sibling, or cousin pair provides once the dependent and once the explanatory variable.[47] While the consistency of the regression estimates for heritability and environmental influences is not affected by double-entry, the standard errors of the coefficients are biased and need to be adjusted.[47] In the present study in the logistic regression models, we used the "variance covariance (vce) cluster" method using STATA, which calculated robust standard errors using families as clusters.[49] The vce (cluster clustvar) specifies that the standard errors allow for intragroup correlation, relaxing the usual requirement that the observations be independent.

Statistical significance was set to p<0,05, all tests were 2-tailed. Data were analyzed using STATA version 16.1 (StataCorp LLC, College Station, TX, USA)

For peer review only

Results

Multimorbidity

A total of 2,694,442 unique individuals were included in the study, 49% of these were females. The mean age at the end of the study was 32 years. During the study period between 1997 and 2015, a total of 440,742 (16.36%) individuals were diagnosed with multimorbidity (Table 1). Of these 253,931 (57.61%) were females and the proportion of females increased with increasing multimorbidity score with 63.27% of the individuals with a score of ≥5 being female (Supplementary Table 2). Multimorbidity was associated with level of education where 25.47% of the multimorbid individuals had an education of ≥12 years compared to 31.32% in the non-multimorbid group (Table 1). Multimorbidity also increased with older age at the end of the study, multimorbid individuals had a mean age of 35 years versus 31 in non-multimorbid groups. Baseline characteristics can be found in Table 1 and in Supplementary Table 2.

Table 1. Descriptive findings for all unique study participants with stratification according to multimorbidity scores for number of unique individuals, sex, education, age at the end of study, birth date, and VTE (venous thromboembolism).

		Multimorbidity scores	
	All	≤ 1	≥ 2
Unique individuals, n (%)	2694442 (100)	2253700 (83.64)	440742 (16.36)
Sex, Female, % (n)	48.73 (1312989)	46.99 (1059058)	57.61 (253931)
Education (≥12 years), % (n)	30.36 (818146)	31.32 (705901)	25.47 (112245)
Age at end of study, Median (IQR) Range (min-max)	32 (22-43) (0 -68)	31 (22-42) (0-67)	35 (24-46) (0-68)
Year of birth, Median (IQR) Range (min-max)	1983 (1972-1993) (1947-2005)	1984 (1973-1993) (1948-2005)	1980 (1969-1990) (1947-2005)
VTE, n (%)	16099 (0.6)	9116 (0.40)	6983 (1.58)

IQR=Interquartile Range.

For peer review only - http://bmjopen.bmj.com/site/about/guidelines.xhtml

VTE

There was a total of 16,099 cases with diagnosed VTE during the study period between 1997 and 2015. In individuals with multimorbidity, 1.58 % had VTE compared to 0.40% in individuals without multimorbidity. Of the female individuals 0.66% had VTE and among males the figure was 0.54%. VTE increased with age with 1.3% in the oldest group and 0.08% in the youngest group. (Tables 1-3).

Table 2. Odds ratio (OR) for VTE (venous thromboembolism) and multimorbidity (score ≥2) for all and stratified according to sex. Odds Ratios (ORs) with 95 % confidence interval (CI) for multimorbidity. Reference with one or no diseases (score=0 or 1). Total observations n=2694442.

	OR (95% CI)			
	No VTE	VTE	Model 1	Model 2
All	433759	6983	3.96 (3.84-4.09)	3.16 (3.06-3.27)
Males	183732	3079	4.52 (4.32-4.74)	3.45 (3.29-3.62)
Females	250027	3904	3.5 (3.35-3.65)	2.91 (2.79-3.04)

Abbreviations: OR, odds ratio. Model 1 is a crude model (univariable). Model 2 is an adjusted model (multivariable), with adjustments for year of birth, county, and educational attainment.

Table 3. Odds ratio for VTE (venous thromboembolism) according to multimorbidity score (0 to ≥2) and age stratified according to interquartile range (IQR1-4). Odds Ratios (ORs) with 95 % confidence interval (CI) for multimorbidity scores. Reference with no diseases (score=0). Total observations n=2694442.

	OR (95% CI)			
Age groups	No VTE	VTE	Model 1	Model 2
IQR1	135989	4032	3.35 (3.21-3.50)	3.14 (3.01-3.29)
IQR2	106001	1857	3.43 (3.24-3.64)	3.14 (2.96-3.34)
IQR3	104897	919	3.48 (3.21-3.78)	3.09 (2.84-3.37)
IQR4	86872	175	3.52 (2.93-4.22)	3.07 (2.56-3.7)

Abbreviations: OR, odds ratio. Model 1 is a crude model (univariable). Model 2 is an adjusted model (multivariable), with adjustments for sex, year of birth, county, and educational attainment. IQR=interquartile range. IQR1 1947-1972, IQR2 1972-1983, IQR3 1983-1993, IQR4 1993-2005.

For peer review only - http://bmjopen.bmj.com/site/about/guidelines.xhtml

VTE and multimorbidity

In the multimorbidity group a total of 6983 of the 440742 (1.58%) individuals were diagnosed with VTE (Table 1). In the group with a multimorbidity score of ≥5 1603 of the 36313 (4.41%) had VTE during the study period (Supplementary Table 2). The OR for VTE in the adjusted model for an individual with multimorbidity was 3.16 (95%CI 3.06 to 3.27) (Table 2). For males with multimorbidity, the OR for VTE was higher than for females (Table 2). Birth year was stratified into quartiles. For individuals born 1947-1972 the OR for VTE with multimorbidity was 3.14 (95%CI: 3.01 to 3.29) in the adjusted model and for individuals born 1993-2005 the OR for VTE with multimorbidity was 3.07 (95%CI 2.56 to 3.7) (Table 3). Thus, there was no strong age dependence regarding the association between multimorbidity and VTE.

Association between increasing multimorbidity score and VTE

The association of increasing multimorbidity score on VTE was investigated (Table 4). It showed an increased OR for increasing score of multimorbidity. OR for a multimorbidity score of two in the adjusted model was 2.93 (95%CI 2.80 to 3.08), 4.07 (95%CI 3.85 to 4.31) for a multimorbidity score of three, 5.46 (95%CI: 5.10 to 5.85) for a multimorbidity score of four, and for a multimorbidity score of five or more the OR was 9.08 (95%CI: 8.56 to 9.64). (Table 4).

Table 4. Odds ratio for VTE (venous thromboembolism) according to multimorbidity score (0 to ≥5). Odds Ratios (ORs) with 95 % confidence interval (CI) for multimorbidity scores. Reference with no diseases (score=0).

	All (n=2694442)			
	OR (95% CI)			
Multimorbidity score	No VTE	VTE	Model 1	Model 2
Score 0	1611205	5008	1[Reference]	1[Reference]

For peer review only - http://bmjopen.bmj.com/site/about/guidelines.xhtml

Score 1	633379	4108	2.09 (2.00-2.18)	1.94 (1.86-2.02)
Score 2	251558	2652	3.39 (3.24-3.56)	2.93 (2.80-3.08)
Score 3	102969	1665	5.20 (4.92-5.50)	4.07 (3.85-4.31)
Score 4	44522	1063	7.68 (7.18-8.21)	5.46(5.10-5.85)
Score ≥5	34710	1603	14.86 (14.03-15.73)	9.08 (8.56-9.64)

Abbreviations: OR, odds ratio. Model 1 is a crude model (univariable). Model 2 is an adjusted model (multivariable), with adjustments for sex, year of birth, county, and educational attainment.

Age and sex stratification

Multimorbidity score results for sex and age stratification are presented in Supplementary Tables S3-S8. A strong association between multimorbidity and VTE could be observed in both sexes and all age groups.

Familial associations between multimorbidity and VTE

There was mostly a weak association between twin, sibling, half-sibling and cousin history of multimorbidity and VTE (Table 5). The ORs in adjusted models for VTE in patients with multimorbidity were 1.89 in twins (95%CI 1.17 to 3.05), 1.18 in siblings (95%CI 1.12 to

For peer review only - http://bmjopen.bmj.com/site/about/guidelines.xhtml

1.24), 1.1 in half-siblings (95%CI 1.03 to 1.18), and 1.07 in cousins (95%CI 1.04 to 1.1) of those diagnosed with index score more than one (Table 5). Among twins, the adjusted OR for VTE in patients with multimorbidity was =0.73 (95%CI 0.04 to 1.35) for twin score=1, OR=1.57 (95%CI 0.81 to 3.05) for twin score=2, OR=1.26 (95%CI 0.48 to 3.31) for twin score=3, OR=1.48 (95%CI 0.44 to 4.98) for twin score=4, and OR=3.98 (95%CI 1.17 to 9.39) for twin score ≥ 5.

For peer review only

Table 5. Familial associations for multimorbidity and venous thromboembolism (VTE). Odds ratio with 95 % confidence interval (CI) for VTE according to multimorbidity (≥ 2 score) or multimorbidity score (0 to ≥5) in relatives. Reference are relatives with no diseases (score=0) for score but no or one disease for multimorbidity (≥ 2 score).

For peer review only - http://bmjopen.bmj.com/site/about/guidelines.xhtml

	Twins (n=24020)		Siblings (n=1546108)		Half-siblings (n=984976)		Cousins (n=6623156)	
	OR (95% CI)		OR (95% CI)		OR (95% CI)		OR (95% CI)	
Relative score	Model 1	Model 2	Model 1	Model 2	Model 1	Model 2	Model 1	Model 2
Score 0	Reference	Reference	Reference	Reference	Reference	Reference	Reference	Reference
Score 1	0.77 (0.42-1.41)	0.73 (0.4-1.35)	1.19 (1.13-1.25)	1.11 (1.06-1.17)	1.12 (1.05-1.19)	1.08 (1.01-1.15)	1.09(1.06-1.12)	1.04(1.02-1.07)
Score 2	1.81 (0.95-3.46)	1.57 (0.81-3.05)	1.37 (1.28-1.46)	1.19 (1.11-1.27)	1.14 (1.05-1.25)	1.07 (0.98-1.17)	1.16(1.11-1.2)	1.07(1.03-1.11)
Score 3	1.86 (0.74-4.68)	1.26 (0.48-3.31)	1.5 (1.37-1.67)	1.18 (1.07-1.3)	1.35 (1.2-1.51)	1.19 (1.07-1.34)	1.21(1.14-1.27)	1.06(1-1.11)
Score 4	2.66 (0.82-8.59)	1.48 (0.44-4.98)	1.89 (1.67-2.15)	1.34 (1.18-1.53)	1.4 (1.19-1.64)	1.17 (1.0-1.37)	1.38(1.28-1.48)	1.14(1.06-1.22)
Score ≥5	9.31 (4.17-20.79)	3.98 (1.69-9.39)	2.16 (1.88-2.48)	1.31 (1.14-1.51)	1.6 (1.36-1.89)	1.2 (1.02-1.41)	1.52(1.41-1.65)	1.16(1.07-1.26)
Multimorbidity defined by two or more diseases compared with no or one disease.								
Score ≥2	2.55 (1.62-4.02)	1.89 (1.17-3.05)	1.43 (1.36-1.51)	1.18 (1.12-1.24)	1.23 (1.15-1.31)	1.1 (1.03-1.18)	1.19(1.16-1.23)	1.07(1.04-1.1)

Abbreviations: OR=odds ratio. The ORs were derived from double entry and the variance covariance (vce) cluster method was used. Model 1 is a crude model. Model 2 is adjusted for sex, year of birth, county, and educational attainment.

For peer review only - http://bmjopen.bmj.com/site/about/guidelines.xhtml

Discussion

The strong association between multimorbidity and VTE is, to the best of our knowledge, a novel finding. However, the study design does not allow any causal inferences to be drawn regarding multimorbidity and VTE. Still, the strong and increasing association with increasing multimorbidity score suggests that future cohort studies might be worthwhile in order to determine whether a possible causal association between multimorbidity and VTE exists.[50] Our present study shows an association between multimorbidity and VTE with an adjusted OR of 3.16. The VTE risk increases with a higher multimorbidity score and is nine times higher in individuals with a multimorbidity score of five or more compared to individuals with no morbidity. Thus, multimorbidity is strongly associated with VTE. Genetic inheritance has been implicated in multimorbidity.[16] However, our data argue against a major genetic association between multimorbidity and VTE due to the mostly weak familial associations. However, there was a significant familial association, which correlated with the degree of relatedness suggesting a possible shared genetic susceptibility to the association between multimorbidity and VTE, though shared environmental exposures also may be involved. A high multimorbidity score in a patient confers similar odds ratios for VTE as strong genetic or acquired risk factors.[1,2,6,10-12] Moroever, multimorbidity has been associated with inflammatory biomarkers and an association between inflammation and VTE has long been recognized.[51-54] Based on these findings, a future dissection of multimorbidity and its relation to VTE in cohort studies might be worthwhile.

The impact of multimorbidity on VTE is stronger in males compared to females and with a higher relationship among males. On the other hand, females have increased multimorbidity and live longer which may give a double burden for the female population in the context risk of VTE, if multimorbidity will turn out to be an important risk factor for VTE.

For peer review only - http://bmjopen.bmj.com/site/about/guidelines.xhtml

The risk of multimorbidity is increasing with age and more individuals worldwide are multimorbid.[16] Our study population is relatively young and confirms previous studies that multimorbidity may also affect young persons.[15] However, there was a strong association with multimorbidity in all age groups suggesting that multimorbidity might also be of importance in older age groups not studied here; this needs to be confirmed in further studies. If multimorbidity in future studies turn out to be a risk factor for VTE, it is possible that multimorbidity is an important contributor to the high incidence of VTE in society due to its high prevalence. Common risk factors, both genetic and acquired, are often seen as individual risk factors in specific settings, like surgery, pregnancy, puerperium, oral contraceptives, surgery and trauma as well as for specific diseases, such as cancer. The present study suggests that we might count the total burden of disease into a multimorbidity score to get a better risk estimate of VTE. However, future prospective studies need to determine which combinations of diseases are most strongly associated with VTE.

Since there is no clear definition of which diagnoses should be used in the field of multimorbidity the ones that are included are sometimes random.[14-19] In this study we used a modified score validated by Barnett et al. 2012.[14] The NCDs that were used in this study are all common. They all have an impact on healthcare, and most are considerably stable during an individual's life and can coexist and create a significant health burden.[14] It will be important to investigate whether a certain combination of diagnoses and multimorbidity has an especially strong association with VTE and further studies are needed to investigate this matter. However, the association between multimorbidity and VTE suggests that not only specific diseases are important but also the total burden of certain diseases.

For peer review only - http://bmjopen.bmj.com/site/about/guidelines.xhtml

Strengths and limitations

The limited number of confounders is a potential limitation. An important limitation of the study is that we did not determine any temporal association between multimorbidity and VTE. We therefore cannot draw any causal inferences or exclude reverse causality. However, only atrial fibrillation and arterial atherosclerotic diseases have been described to exhibit a bidirectional association with VTE.[36-38] Moreover, it is possible that certain combinations of diseases are more strongly associated with VTE than others. It will be of importance to further dissect the occurrence of multimorbidity and its relation to future risk for VTE in cohort studies. The study period was chosen to minimize the risk of missed data and to increase the coverage of individuals. Furthermore, having more comprehensive ICD-10 codes decreases the risk of inaccurate coding from the registers. A strength of the study is the high coverage and high validity of the registers and the large number of unique individuals.[41-46] A limitation is that only Swedish-born individuals born by Swedish parents were included. It therefore remains for a future study to show if the results may be generalized to other populations. Another limitation is that the data did not include biological and environmental risk factors such as smoking, alcohol consumption, height, weight, blood pressure and exercise habits. To make up for the absence of such data we adjusted for educational level, which is related to lifestyle factors.[55] Also, the included patients were relatively young with an age-range of 0-68 and a mean of 32 at the end of the study and generalization of the elderly is uncertain, even though within this age span between 0-68 years there was no age effect on the association. Hence there is a need for further studies in the future.

For peer review only - http://bmjopen.bmj.com/site/about/guidelines.xhtml

Conclusion

We have shown that an association exists between multimorbidity and multimorbidity severity (i.e. number of NCDs) and venous thromboembolism. Weak shared familial factors (environmental or genetic) may also be involved. Multimorbidity might be worthwhile to further dissect in relation to VTE in future cohort studies in order to study a temporal and possibly causal association.

Contributorship statement

JA, MP, BH, JS, AH, KS and BZ contributed to the conception and design of the study; JS and KS contributed to the acquisition of data; JA, MP, BH, JS, AH, KS and BZ contributed to the analysis and interpretation of data; JA drafted the manuscript; and JA, MP, BH, JS, AH, KS and BZ revised it critically and approved the final version. JA, MP, BH, JS, AH, KS and BZ had full access to all the data (including statistical reports and tables) and take responsibility for the integrity of the data and the accuracy of their analysis.

Competing interests

The authors have nothing to disclose.

Funding

ALF-funding from Region Skåne (N/A) and the Swedish Research Council (2020-01824).

Data sharing statement

We are not allowed to share our data as the data are drawn from registries owned by a third party, i.e. the Swedish authorities. The reason behind this is that public availability could potentially compromise patient confidentiality or privacy. Permission to use the data is issued

For peer review only

For peer review only - http://bmjopen.bmj.com/site/about/guidelines.xhtml

to researchers (if certain conditions are fulfilled) by the National Board of Health and Welfare and by Statistics Sweden. Request to use the data can be applied for from the Swedish National Board of Health and Welfare (https://www.socialstyrelsen.se/en/statistics-and-data/statistics/statistical-database/) and Statistics Sweden (https://www.scb.se/en/About-us/contact-us/). An application for use of the same minimal dataset, which we have used in this study, should include a motivation, a referral to the present study and an addition of a list of variables, before submission of a data request to the Swedish National Board of Health and Welfare and Statistics Sweden using the contact addresses above. Then the authorities will coordinate the handling of the request and do a special review.

Ethics statement

This study was approved by the Regional Ethical Review Board in Lund (2012/795 and later amendments).

Acknowledgments

We thank Patrick Reilly, science editor at Center for Primary Health Care Research, Department of Clinical Science, Lund University and Region Skåne, Malmö, Sweden, for language review.

References

1. White RH. The epidemiology of venous thromboembolism. Circulation. 2003;107(23 Suppl 1):I4-8.
2. Heit JA. Epidemiology of venous thromboembolism. Nat Rev Cardiol. 2015;12:464-74.
3. Hansson PO, Welin L, Tibblin G, Eriksson H. Deep vein thrombosis and pulmonary embolism in the general population. 'The Study of Men Born in 1913'. Arch Intern Med. 1997;157(15):1665-70.
4. Manderstedt E, Halldén C, Lind-Halldén C, Elf J, Svensson PJ, Engström G, Melander O, Baras A, Lotta LA, Zöller B; Regeneron Genetics Center. Thrombomodulin (THBD) gene variants and thrombotic risk in a population-based cohort study. J Thromb Haemost. 2022;20(4):929-935.

For peer review only - http://bmjopen.bmj.com/site/about/guidelines.xhtml

5. Naess IA, Christiansen SC, Romundstad P, Cannegieter SC, Rosendaal FR, Hammerstrøm J. Incidence and mortality of venous thrombosis: a population-based study. J Thromb Haemost. 2007;5(4):692-9.
6. Rosendaal FR. Venous thrombosis: a multicausal disease. Lancet. 1999;353(9159):1167-73.
7. Brandjes DP, Büller HR, Heijboer H, Huisman MV, de Rijk M, Jagt H, ten Cate JW. Randomised trial of effect of compression stockings in patients with symptomatic proximal-vein thrombosis. Lancet. 1997;349(9054):759-62.
8. Anderson FA Jr, Wheeler HB, Goldberg RJ, Hosmer DW, Patwardhan NA, Jovanovic B, Forcier A, Dalen JE. A population-based perspective of the hospital incidence and case-fatality rates of deep vein thrombosis and pulmonary embolism. The Worcester DVT Study. Arch Intern Med. 1991;151(5):933-8.
9. Zöller B, Pirouzifard M, Sundquist J, Sundquist K. Association of Short-Term Mortality of Venous Thromboembolism with Family History of Venous Thromboembolism and Charlson Comorbidity Index. Thromb Haemost. 2019;119(1):48-55.
10. Zöller B, Svensson PJ, Dahlbäck B, Lind-Hallden C, Hallden C, Elf J. Genetic risk factors for venous thromboembolism. Expert Rev Hematol. 2020;13(9):971-981.
11. Manderstedt E, Manderstedt E, Lind-Halldén C, Halldén C, Elf J, Svensson PJ, Dahlbäck B, Engström G, Melander O, Baras A, Lotta LA, Zöller B; Regeneron Genetics Center 4 [Link]. Classic Thrombophilias and Thrombotic Risk Among Middle-Aged and Older Adults: A Population-Based Cohort Study. J Am Heart Assoc. 2022;11(4):e023018.
12. Lijfering WM, Rosendaal FR, Cannegieter SC. Risk factors for venous thrombosis - current understanding from an epidemiological point of view. Br J Haematol. 2010;149(6):824-33.
13. Zöller B, Li X, Sundquist J, Sundquist K. Risk of pulmonary embolism in patients with autoimmune disorders: a nationwide follow-up study from Sweden. Lancet. 2012;379(9812):244-9.
14. Barnett K, Mercer SW, Norbury M, Watt G, Wyke S, Guthrie B. Epidemiology of multimorbidity and implications for health care, research, and medical education: a cross-sectional study. Lancet. 2012;380(9836):37-43.
15. Troelstra SA, Straker L, Harris M, Brown S, van der Beek AJ, Coenen P. Multimorbidity is common among young workers and related to increased work absenteeism and presenteeism: results from the population-based Raine Study cohort. Scand J Work Environ Health. 2020;46(2):218-227.
16. Dong G, Feng J, Sun F, Chen J, Zhao XM. A global overview of genetically interpretable multimorbidities among common diseases in the UK Biobank. Genome Med. 2021;13(1):110.
17. Johnston MC, Crilly M, Black C, Prescott GJ, Mercer SW. Defining and measuring multimorbidity: a systematic review of systematic reviews. Eur J Public Health. 2019 Feb 1;29(1):182-189.
18. Pearson-Stuttard J, Ezzati M, Gregg EW. Multimorbidity-a defining challenge for health systems. Lancet Public Health. 2019;4(12):e599-e600.
19. Wallace E, Salisbury C, Guthrie B, Lewis C, Fahey T, Smith SM. Managing patients with multimorbidity in primary care. BMJ. 2015;350:h176.

For peer review only - http://bmjopen.bmj.com/site/about/guidelines.xhtml

20. Wang Y, Ding C, Guo C, Wang J, Liu S. Association between thyroid dysfunction and venous thromboembolism: A systematic review and meta-analysis. Medicine (Baltimore). 2023;102(11):e33301.
21. Guo Y, Zhou F, Xu H. Gout and risk of venous thromboembolism: A systematic review and meta-analysis of cohort studies. Int J Rheum Dis. 2023;26(2):344-353.
22. Allman-Farinelli MA. Obesity and venous thrombosis: a review. Semin Thromb Hemost. 2011;37(8):903-7.
23. Zhang XY, Dong HC, Wang WF, Zhang Y. Risk of venous thromboembolism in children and adolescents with inflammatory bowel disease: A systematic review and meta-analysis. World J Gastroenterol. 2022;28(16):1705-1717.
24. Kowal C, Peyre H, Amad A, Pelissolo A, Leboyer M, Schürhoff F, Pignon B. Psychotic, Mood, and Anxiety Disorders and Venous Thromboembolism: A Systematic Review and Meta-Analysis. Psychosom Med. 2020;82(9):838-849.
25. Tang L, Wu YY, Lip GY, Yin P, Hu Y. Heart failure and risk of venous thromboembolism: a systematic review and meta-analysis. Lancet Haematol. 2016;3(1):e30-44.
26. Bai J, Ding X, Du X, Zhao X, Wang Z, Ma Z. Diabetes is associated with increased risk of venous thromboembolism: a systematic review and meta-analysis. Thromb Res. 2015;135(1):90-5.
27. Gariani K, Mavrakanas T, Combescure C, Perrier A, Marti C. Is diabetes mellitus a risk factor for venous thromboembolism? A systematic review and meta-analysis of case-control and cohort studies. Eur J Intern Med. 2016;28:52-8.
28. Bell EJ, Folsom AR, Lutsey PL, Selvin E, Zakai NA, Cushman M, Alonso A. Diabetes mellitus and venous thromboembolism: A systematic review and meta-analysis. Diabetes Res Clin Pract. 2016;111:10-8.
29. Ahmed O, Geraldes R, DeLuca GC, Palace J. Multiple sclerosis and the risk of systemic venous thrombosis: A systematic review. Mult Scler Relat Disord. 2019;27:424-430.
30. Ungprasert P, Sanguankeo A, Upala S, Suksaranjit P. Psoriasis and risk of venous thromboembolism: a systematic review and meta-analysis. QJM. 2014;107(10):793-7.
31. Horsted F, West J, Grainge MJ. Risk of venous thromboembolism in patients with cancer: a systematic review and meta-analysis. PLoS Med. 2012;9(7):e1001275.
32. Qi X, Ren W, Guo X, Fan D. Epidemiology of venous thromboembolism in patients with liver diseases: a systematic review and meta-analysis. Intern Emerg Med. 2015;10(2):205-17.
33. Hu LJ, Ji B, Fan HX. Venous thromboembolism risk in rheumatoid arthritis patients: a systematic review and updated meta-analysis. Eur Rev Med Pharmacol Sci. 2021;25(22):7005-7013.
34. Bello N, Meyers KJ, Workman J, Marcano Belisario J, Cervera R. Systematic Literature Review and Meta-analysis of Venous Thromboembolism Events in Systemic Lupus Erythematosus. Rheumatol Ther. 2023;10(1):7-34.
35. Zöller B, Pirouzifard M, Memon AA, Sundquist J, Sundquist K. Risk of pulmonary embolism and deep venous thrombosis in patients with asthma: a nationwide case-control study from Sweden. Eur Respir J. 2017;49(2):1601014
36. Bikdeli B, Abou Ziki MD, Lip GYH. Pulmonary Embolism and Atrial Fibrillation: Two Sides of the Same Coin? A Systematic Review. Semin Thromb Hemost. 2017;43(8):849-863.

For peer review only - http://bmjopen.bmj.com/site/about/guidelines.xhtml

For peer review only

37. Prandoni P, Bilora F, Marchiori A, Bernardi E, Petrobelli F, Lensing AW, Prins MH, Girolami A. An association between atherosclerosis and venous thrombosis. N Engl J Med. 2003;348(15):1435-41.
38. Becattini C, Vedovati MC, Ageno W, Dentali F, Agnelli G. Incidence of arterial cardiovascular events after venous thromboembolism: a systematic review and a meta-analysis. J Thromb Haemost. 2010;8(5):891-7.
39. Chen TL, Lee LL, Huang HK, Chen LY, Loh CH, Chi CC. Association of Risk of Incident Venous Thromboembolism With Atopic Dermatitis and Treatment With Janus Kinase Inhibitors: A Systematic Review and Meta-analysis. JAMA Dermatol. 2022;158(11):1254-1261.
40. Risch N. The genetic epidemiology of cancer: interpreting family and twin studies and their implications for molecular genetic approaches. Cancer Epidemiol Biomarkers Prev. 2001;10:733-41
41. Rosen M, Hakulinen T. Use of disease registers. In: Ahrens W, Pigeot I, eds. Handbook of Epidemiology. Springer-Verlag; 2005:231–252.
42. Ludvigsson JF, Otterblad-Olausson P, Pettersson BU, Ekbom A. The Swedish personal identity number: possibilities and pitfalls in healthcare and medical research. Eur J Epidemiol. 2009;24(11):659-67.
43. Ludvigsson JF, Andersson E, Ekbom A, Feychting M, Kim JL, Reuterwall C, Heurgren M, Olausson PO. External review and validation of the Swedish national inpatient register. BMC Public Health. 2011;11:450.
44. Ludvigsson JF, Almqvist C, Bonamy AK, Ljung R, Michaëlsson K, Neovius M, Stephansson O, Ye W. Registers of the Swedish total population and their use in medical research. Eur J Epidemiol. 2016;31(2):125-36.
45. Ekbom A. The Swedish Multi-generation Register. Methods Mol Biol. 2011;675:215-20.
46. Zöller B. Nationwide family studies of cardiovascular diseases—clinical and genetic implications of family history. EMJ Cardiol. 2013;1:102–113.
47. Kohler HP, Rodgers JL. DF-analyses of heritability with double-entry twin data: asymptotic standard errors and efficient estimation. Behav Genet. 2001 Mar;31(2):179-91.
48. Rosengren A, Fredén M, Hansson PO, Wilhelmsen L, Wedel H, Eriksson H. Psychosocial factors and venous thromboembolism: a long-term follow-up study of Swedish men. J Thromb Haemost. 2008;6(4):558-64.
49. McLoughlin G, Palmer JA, Rijsdijk F, Makeig S. Genetic overlap between evoked frontocentral theta-band phase variability, reaction time variability, and attention-deficit/hyperactivity disorder symptoms in a twin study. Biol Psychiatry. 2014; 75: 238-47.
50. Hill AB. The environment and disease: association or causation? Proc R Soc Med. 1965;58(5):295-300.
51. St Sauver J, Rocca W, LeBrasseur N, Chamberlain A, Olson J, Jacobson D, McGree M, Mielke M. Inflammatory biomarkers, multi-morbidity, and biologic aging. J Int Med Res. 2022;50(7):3000605221109393.
52. Fabbri E, An Y, Zoli M, Simonsick EM, Guralnik JM, Bandinelli S, Boyd CM, Ferrucci L. Aging and the burden of multimorbidity: associations with inflammatory and anabolic hormonal biomarkers. J Gerontol A Biol Sci Med Sci. 2015;70(1):63-70.

For peer review only - http://bmjopen.bmj.com/site/about/guidelines.xhtml

53. Wessman T, Tofik R, Ruge T, Melander O. Associations between biomarkers of multimorbidity burden and mortality risk among patients with acute dyspnea. Intern Emerg Med. 2022;17(2):559-567.
54. Colling ME, Tourdot BE, Kanthi Y. Inflammation, Infection and Venous Thromboembolism. Circ Res. 2021;128(12):2017-2036
55. Winkleby MA, Jatulis DE, Frank E, Fortmann SP. Socioeconomic status and health: how education, income, and occupation contribute to risk factors for cardiovascular disease. Am J Public Health. 1992;82(6):816-20.

For peer review only

For peer review only - http://bmjopen.bmj.com/site/about/guidelines.xhtml

Supplementary Appendix: Authors: Ahrén J, Pirouzifard M, Holmquist B, Sundquist J, Halling A, Sundquist K, Zöller B

Table of content pages 1-9 **Pages**

1. Supplementary Table S1 2-3
2. Supplementary Table S2 4
3. Supplementary Table S3 5
4. Supplementary Table S4 6
5. Supplementary Table S5 7
6. Supplementary Table S6 8
7. Supplementary Table S7 9
8. Supplementary Table S8 10

For peer review only

Supplementary Table S1**.** Diseases with ICD codes included in the multimorbidity score modified and adapted to Swedish ICD-10 codes after Barnett K et al (2012)*. Infectious diseases, i.e. viral hepatitis, were excluded and six common non-communicable diseases (NCDs) were added: dermatitis or eczema, gout, arthrosis, osteoporosis, obesity, and pancreatitis. One point for each disease. The theoretical range of the score is 0-45 points.

	Included disorders in the multimorbidity score	ICD-10**
1	Hypertension	I10-I15
2	Affective disorders	F30-F39
3	Painful back condition	M50-54
4	Asthma	J45-J46
5	CHD	I20-I25
6	Ulcer disease	K221, K25-K28
7	Diabetes	E10-E14
8	Thyroid disorders	E00-E07
9	Connective tissue disease	M05, M06, M08, M09, M30-M36, D86
10	Hearing loss (or impaired)	H90-H91
11	Chronic obstructive pulmonary disease (COPD)	J41-J44
12	Anxiety	F40-F48
13	Irritable bowel syndrome (IBS)	K58
14	Cancer	C00-C99
15	Alcohol use disorders	F10
16	Psychoactive substance misuse	F11-F14, F16, F18, F19
17	Constipation	K590
18	Cerebrovascular disease	I60-I69
19	Renal disease	N00-N19, Q61
20	Diverticular disease of intestine	K57
21	Atrial fibrillation	I48
22	Atherosclerosis	I70-I74, I77
23	Heart failure	I50, I110, I130, I132
24	Prostate disease	N40-N42
25	Glaucoma	H40, H42

For peer review only - http://bmjopen.bmj.com/site/about/guidelines.xhtml

26	Epilepsy	G40, G41
27	Dementia	F00-F03
28	Schizophrenia disorders	F20, F21
29	Psoriasis	L40
30	Dermatitis or Eczema	L20-L30
31	Inflammatory bowel disease (IBD)	K50, K51
32	Migraine	G43, G440, G441
33	Blindness & low vision	H53-H54
34	Chronic sinusitis	J32
35	Learning disability	F81
36	Anorexia or bulimia	F50
37	Bronchiectasis	J47
38	Parkinson's disease	G20, G21, G22
39	Multiple sclerosis	G35
40	Liver disease	K70-K77
41	Gout	M10
42	Arthrosis	M15-M19
43	Osteoporosis	M80-M82
44	Obesity	E65, E66
45	Pancreas diseases	K85, K86

*Barnett K, Mercer SW, Norbury M, Watt G, Wyke S, Guthrie B. Epidemiology of multimorbidity and implications for health care, research, and medical education: a cross-sectional study. Lancet 2012; 380: 37-43.

**ICD=International classification of disease.

For peer review only

For peer review only - http://bmjopen.bmj.com/site/about/guidelines.xhtml

Supplementary Table 2. Descriptive findings for all unique study participants with stratification according to multimorbidity scores for number of unique individuals, sex, education, age at the end of study, birth date, and VTE (venous thromboembolism).

		Multimorbidity scores							
	All	0	1	2	3	4	≥ 5	≤ 1	≥ 2
Unique individuals, n (%)	2694442 (100)	1616213 (59.98)	637487 (23.66)	254210 (9.43)	104634 (3.88)	45585 (1.69)	36313 (1.35)	2253700 (83.64)	440742 (16.36)
Sex, Female, % (n)	48.73 (1312989)	45.25 (731337)	51.41 (327721)	55.66 (141488)	59.07 (61808)	60.68 (27661)	63.27 (22974)	46.99 (1059058)	57.61 (253931)
Education (≥12 years), % (n)	30.36 (818146)	31.97 (516774)	29.67 (189127)	27.16 (69050)	24.80 (25946)	22.45 (10232)	19.32 (7017)	31.32 (705901)	25.47 (112245)
Age at end of study, Median (IQR) Range (min-max)	32 (22-43) (0 -68)	31 (22-42) (0-67)	32 (22-43) (0-67)	33 (23-45) (0-67)	36 (25-46) (1-66)	38 (27-48) (1-67)	42 (31-50) (3-68)	31 (22-42) (0-67)	35 (24-46) (0-68)
Year of birth, Median (IQR) Range (min-max)	1983 (1972-1993) (1947-2005)	1984 (1973-1993) (1948-2005)	1983 (1972-1993) (1948-2005)	1981 (1970-1992) (1948-2005)	1979 (1968-1990) (1949-2005)	1977 (1967-1988) (1948-2005)	1972 (1964-1984) (1947-2005)	1984 (1973-1993) (1948-2005)	1980 (1969-1990) (1947-2005)
VTE, n (%)	16099 (0.6)	5008 (0.31)	4108 (0.64)	2652 (1.04)	1665 (1.59)	1063 (2.33)	1603 (4.41)	9116 (0.40)	6983 (1.58)

IQR=Interquartile Range.

Supplementary Table S3. Odds ratio for VTE according to multimorbidity score (0 to ≥5) in males. Odds Ratios (ORs) with 95 % confidence interval (CI) for multimorbidity scores. Reference with no diseases (score=0).				
	Male (n=1381453)			
	OR (95% CI)			
VTE score	No VTE	VTE	Model 1	Model 2
Score 0	882367	2509	1[Reference]	1[Reference]
Score 1	307866	1900	2.17 (2.04-2.30)	2.03 (1.91-2.26)
Score 2	111533	1189	3.75 (3.5-4.02)	3.20 (2.99-3.44)
Score 3	42063	763	6.38 (5.88-6.92)	4.73 (4.35-5.14)
Score 4	17441	483	9.74 (8.83-10.75)	6.3(5.69-6.96)
Score ≥5	12695	644	17.84 (16.33-19.49)	9.38 (8.55-10.28)

Abbreviations: OR, odds ratio. Model 1 is a crude model (univariable). Model 2 is an adjusted model (multivariable), with adjustments for year of birth, county, and educational attainment.

For peer review only

Supplementary Table S4. Odds ratio for VTE according to multimorbidity score (0 to ≥5) in females. Odds Ratios (ORs) with 95 % confidence interval (CI) for multimorbidity scores. Reference with no diseases (score=0).

	Female (n=1312989)			
	OR (95% CI)			
VTE score	No VTE	VTE	Model 1	Model 2
Score 0	728838	2499	1[Reference]	1[Reference]
Score 1	325513	2208	1.98 (1.88-2.10)	1.84 (1.73-1.94)
Score 2	140025	1463	3.05 (2.86-3.25)	2.67 (2.5-2.85)
Score 3	60906	902	4.32 (4.0-4.66)	3.53 (3.26-3.81)
Score 4	27081	580	6.25 (5.70-6.84)	4.76 (4.34-5.22)
Score ≥5	22015	959	12.70 (11.78-13.70)	8.58 (7.94-9.27)

Abbreviations: OR, odds ratio. Model 1 is a crude model (univariable). Model 2 is an adjusted model (multivariable), with adjustments for year of birth, county, and educational attainment.

For peer review only

Supplementary Table S5. Odds ratio for VTE according to multimorbidity score (0 to ≥5) for individuals born 1947-1972. Odds Ratios (ORs) with 95 % confidence interval (CI) for multimorbidity scores. Reference with no diseases (score=0).

	All (n=641211)			
	OR (95% CI)			
VTE score	NO VTE	VTE	Model 1	Model 2
Score 0	340353	2331	1[Reference]	1[Reference]
Score 1	156444	2062	1.92 (1.78-2.00)	1.89 (1.78-2.00)
Score 2	70005	1437	3 (2.81-3.2)	2.89 (2.7-3.08)
Score 3	33176	957	4.22 (3.9-4.54)	4 (3.7-4.31)
Score 4	16542	624	5.51 (5.04-6.02)	5.13 (4.68-5.62)
Score ≥5	16266	1014	9.1 (8.44-9.81)	8.3 (7.68-8.97)

Abbreviations: OR, odds ratio. Model 1 is a crude model (univariable). Model 2 is an adjusted model (multivariable), with adjustments for sex, year of birth, county, and educational attainment.

For peer review only

Supplementary Table S6. Odds ratio for VTE according to multimorbidity score (0 to ≥5) for individuals born 1972-1983. Odds Ratios (ORs) with 95 % confidence interval (CI) for multimorbidity scores. Reference with no diseases (score=0).

	All (n=682582)			
	OR (95% CI)			
VTE score	NO VTE	VTE	Model 1	Model 2
Score 0	415987	1638	1[Reference]	1[Reference]
Score 1	155822	1277	2.08 (1.93-2.24)	2.01 (1.87-2.16)
Score 2	60801	725	3.02 (2.77-3.31)	2.85 (2.61-3.11)
Score 3	25237	468	4.71 (4.25-5.22)	4.33 (3.9-4.81)
Score 4	11184	280	6.36 (5.59-7.23)	5.74 (5.04-6.54)
Score ≥5	8779	384	11.11 (9.92-12.44)	9.7 (8.64-10.89)

Abbreviations: OR, odds ratio. Model 1 is a crude model (univariable). Model 2 is an adjusted model (multivariable), with adjustments for sex, year of birth, county, and educational attainment.

review only

Supplementary Table S7. Odds ratio for VTE according to multimorbidity score (0 to ≥5) for individuals born 1983-1993. Odds Ratios (ORs) with 95 % confidence interval (CI) for multimorbidity scores. Reference with no diseases (score=0).

	All (n=691267)			
	OR (95% CI)			
VTE score	NO VTE	VTE	Model 1	Model 2
Score 0	427804	845	1[Reference]	1[Reference]
Score 1	156178	624	2.02 (1.82-2.24)	1.94 (1.75-2.15)
Score 2	61537	399	3.28 (2.91-3.7)	3.04 (2.69-3.43)
Score 3	25768	204	4.01 (3.44-4.67)	3.59 (3.07-4.2)
Score 4	10734	137	6.46 (5.39-7.75)	5.63 (4.68-6.78)
Score ≥5	6858	179	13.21 (11.23-15.55)	10.91 (9.21-12.91)

Abbreviations: OR, odds ratio. Model 1 is a crude model (univariable). Model 2 is an adjusted model (multivariable), with adjustments for sex, year of birth, county, and educational attainment.

review only

Supplementary Table S8. Odds ratio for VTE according to multimorbidity score (0 to ≥5) for individuals born 1993-2005. Odds Ratios (ORs) with 95 % confidence interval (CI) for multimorbidity scores. Reference with no diseases (score=0).

	All (n=679382)			
	OR (95% CI)			
VTE score	NO VTE	VTE	Model 1	Model 2
Score 0	427061	194	1[Reference]	1[Reference]
Score 1	164935	145	1.94 (1.56-2.4)	1.94 (1.56-2.41)
Score 2	59215	91	3.38 (2.64-4.34)	3.16 (2.46-4.06)
Score 3	18788	36	4.22 (2.95-6.02)	3.49 (2.44-5)
Score 4	6062	22	7.99 (5.14-12.43)	5.92 (3.8-9.24)
Score ≥5	2807	26	20.4 (13.52-30.76)	13.46 (8.87-20.42)

Abbreviations: OR, odds ratio. Model 1 is a crude model (univariable). Model 2 is an adjusted model (multivariable), with adjustments for sex, year of birth, county, and educational attainment.

For peer review only - http://bmjopen.bmj.com/site/about/guidelines.xhtml

STROBE 2007 (v4) Statement—Checklist of items that should be included in reports of *cross-sectional studies*

Section/Topic	Item #	Recommendation	Reported on page #
**Title and abstract**	1	(*a*) Indicate the study's design with a commonly used term in the title or the abstract	1-3
		(*b*) Provide in the abstract an informative and balanced summary of what was done and what was found	2-3
**Introduction**			
Background/rationale	2	Explain the scientific background and rationale for the investigation being reported	5-7
Objectives	3	State specific objectives, including any prespecified hypotheses	5-7
**Methods**			
Study design	4	Present key elements of study design early in the paper	7-8
Setting	5	Describe the setting, locations, and relevant dates, including periods of recruitment, exposure, follow-up, and data collection	7-8
Participants	6	(*a*) Give the eligibility criteria, and the sources and methods of selection of participants	7-8
Variables	7	Clearly define all outcomes, exposures, predictors, potential confounders, and effect modifiers. Give diagnostic criteria, if applicable	8-9
Data sources/ measurement	8*	For each variable of interest, give sources of data and details of methods of assessment (measurement). Describe comparability of assessment methods if there is more than one group	8-9
Bias	9	Describe any efforts to address potential sources of bias	*9-10*
Study size	10	Explain how the study size was arrived at	7-10
Quantitative variables	11	Explain how quantitative variables were handled in the analyses. If applicable, describe which groupings were chosen and why	7-10
Statistical methods	12	(*a*) Describe all statistical methods, including those used to control for confounding	9-10
		(*b*) Describe any methods used to examine subgroups and interactions	9-10
		(*c*) Explain how missing data were addressed	9-10
		(*d*) If applicable, describe analytical methods taking account of sampling strategy	9-10
		(*e*) Describe any sensitivity analyses	9-10
**Results**			

For peer review only

Participants	13*	(a) Report numbers of individuals at each stage of study—eg numbers potentially eligible, examined for eligibility, confirmed eligible, included in the study, completing follow-up, and analysed	11-16
		(b) Give reasons for non-participation at each stage	NA -nationwide study
		(c) Consider use of a flow diagram	Not used
Descriptive data	14*	(a) Give characteristics of study participants (eg demographic, clinical, social) and information on exposures and potential confounders	11-16
		(b) Indicate number of participants with missing data for each variable of interest	NA -nationwide study
Outcome data	15*	Report numbers of outcome events or summary measures	11-16
Main results	16	(*a*) Give unadjusted estimates and, if applicable, confounder-adjusted estimates and their precision (eg, 95% confidence interval). Make clear which confounders were adjusted for and why they were included	11-16
		(*b*) Report category boundaries when continuous variables were categorized	11-16
		(*c*) If relevant, consider translating estimates of relative risk into absolute risk for a meaningful time period	11-16
Other analyses	17	Report other analyses done—eg analyses of subgroups and interactions, and sensitivity analyses	11-16
**Discussion**			
Key results	18	Summarise key results with reference to study objectives	17-20
Limitations	19	Discuss limitations of the study, taking into account sources of potential bias or imprecision. Discuss both direction and magnitude of any potential bias	17-20
Interpretation	20	Give a cautious overall interpretation of results considering objectives, limitations, multiplicity of analyses, results from similar studies, and other relevant evidence	17-20
Generalisability	21	Discuss the generalisability (external validity) of the study results	17-20
**Other information**			
Funding	22	Give the source of funding and the role of the funders for the present study and, if applicable, for the original study on which the present article is based	20

*Give information separately for cases and controls in case-control studies and, if applicable, for exposed and unexposed groups in cohort and cross-sectional studies.

Note: An Explanation and Elaboration article discusses each checklist item and gives methodological background and published examples of transparent reporting. The STROBE checklist is best used in conjunction with this article (freely available on the Web sites of PLoS Medicine at http://www.plosmedicine.org/, Annals of Internal Medicine at http://www.annals.org/, and Epidemiology at http://www.epidem.com/). Information on the STROBE Initiative is available at www.strobe-statement.org.

For peer review only

For peer review only

A hypothesis - generating Swedish extended national cross-sectional family study of multimorbidity severity and venous thromboembolism

Journal:	*BMJ Open*
Manuscript ID	bmjopen-2023-072934.R2
Article Type:	Original research
Date Submitted by the Author:	29-May-2023

Complete List of Authors:	Ahrén, Jonatan; Lund University, Center for Primary Health Care Research<br>Pirouzifard, Mirnabi; Lund University, Center for Primary Health Care Research<br>Holmquist, Björn; Lund University, Department of Statistics<br>Sundquist, Jan; Lund University, Center for Primary Health Care Research<br>Halling, Anders; Lund University, Center for Primary Health Care Research<br>Sundquist, Kristina; Lund University, Center for Primary Health Care Research<br>Zöller, Bengt; Lund University, Center for Primary Health Care Research

**Primary Subject Heading**:	Cardiovascular medicine
Secondary Subject Heading:	Epidemiology, General practice / Family practice
Keywords:	Thromboembolism < CARDIOLOGY, EPIDEMIOLOGY, GENERAL MEDICINE (see Internal Medicine), Vascular medicine < INTERNAL MEDICINE, Primary Health Care

I, the Submitting Author has the right to grant and does grant on behalf of all authors of the Work (as defined in the below author licence), an exclusive licence and/or a non-exclusive licence for contributions from authors who are: i) UK Crown employees; ii) where BMJ has agreed a CC-BY licence shall apply, and/or iii) in accordance with the terms applicable for US Federal Government officers or employees acting as part of their official duties; on a worldwide, perpetual, irrevocable, royalty-free basis to BMJ Publishing Group Ltd ("BMJ") its licensees and where the relevant Journal is co-owned by BMJ to the co-owners of the Journal, to publish the Work in this journal and any other BMJ products and to exploit all rights, as set out in our licence.

The Submitting Author accepts and understands that any supply made under these terms is made by BMJ to the Submitting Author unless you are acting as an employee on behalf of your employer or a postgraduate student of an affiliated institution which is paying any applicable article publishing charge ("APC") for Open Access articles. Where the Submitting Author wishes to make the Work available on an Open Access basis (and intends to pay the relevant APC), the terms of reuse of such Open Access shall be governed by a Creative Commons licence – details of these licences and which Creative Commons licence will apply to this Work are set out in our licence referred to above.

Other than as permitted in any relevant BMJ Author's Self Archiving Policies, I confirm this Work has not been accepted for publication elsewhere, is not being considered for publication elsewhere and does not duplicate material already published. I confirm all authors consent to publication of this Work and authorise the granting of this licence.

For peer review only

A hypothesis - generating Swedish extended national cross-sectional family study of multimorbidity severity and venous thromboembolism

[1]Jonatan Ahrén MD, [1]MirNabi Pirouzifard, PhD; [2]Björn Holmquist, PhD; [1]Jan Sundquist, MD, PhD; [1]Anders Halling PhD, MD; [1]Kristina Sundquist, MD, PhD, [1]Bengt Zöller MD, PhD.

[1]Center for Primary Health Care Research, Lund University/Region Skåne, Malmö, Sweden.

[2]Department of Statistics Lund University Lund Sweden

For peer review only

Running Title: Multimorbidity and venous thromboembolism

Correspondence:

Dr Jonatan Ahrén

Center for Primary Health Care Research

CRC, Building 28, Floor 11

Jan Waldenströms gata 35

Skåne University Hospital

S-205 02 Malmö, Sweden

Tel: +46 735127050; Fax: +46 40 391370

jonatan.ahren@med.lu.se

Word count: 3942 including tables **Abstract word count:** 300

Keywords: epidemiology, genetics medical, medicine, multimorbidity, public health, venous thromboembolism

For peer review only - http://bmjopen.bmj.com/site/about/guidelines.xhtml

Abstract

Objectives: Venous thromboembolism (VTE) is a common worldwide disease. The burden of multimorbidity, i.e. two or more chronic diseases, has increased. Whether multimorbidity is associated with VTE risk remains to be studied. Our aim was to determine any association between multimorbidity and VTE and any possible shared familial susceptibility.

Design: A nationwide extended cross-sectional hypothesis - generating family study between 1997-2015.

Setting: The Swedish Multi-Generation Register, the National Patient Register, the Total Population Register and the Swedish cause of death register were linked.

Participants: 2,694,442 unique individuals were analyzed for VTE and multimorbidity.

Main Outcomes and Measures: Multimorbidity was determined by a counting method using 45 non-communicable diseases (NCD). Multimorbidity was defined by the occurrence of ≥2 diseases. A multimorbidity score was constructed defined by, 0, 1, 2, 3, 4 or 5 or more diseases.

Results: Sixteen percent (n=440742) of the study population was multimorbid. Of the multimorbid patients 58 % were females. There was an association between multimorbidity and VTE. The adjusted odds ratio (OR) for VTE in individuals with multimorbidity (2≥diagnoses) was 3.16 (95%CI 3.06-3.27) compared to individuals without multimorbidity. There was an association between number of diseases and VTE. The adjusted OR was 1.94 (95%CI 1.86-2.02) for one disease, 2.93 (95%CI 2.80-3.08) for two diseases, 4.07 (95%CI 3.85-4.31) for three diseases, 5.46 (95%CI 5.10-5.85) for four diseases, and 9.08 (95%CI 8.56-9.64) for 5≥ diseases. The association between multimorbidity and VTE was stronger in males OR 3.45 (3.29-3.62) than in females OR 2.91 (2.77-3.04). There were significant but mostly weak familial associations between multimorbidity in relatives and VTE.

For peer review only

Conclusions: Increasing multimorbidity exhibits a strong and increasing association with VTE. Familial associations suggest a weak shared familial susceptibility. The association between multimorbidity and VTE suggests that future cohort studies where multimorbidity is used to predict VTE might be worthwhile.

For peer review only

For peer review only - http://bmjopen.bmj.com/site/about/guidelines.xhtml

Strength and limitations of this study

- High coverage and high validity of the registers and the large number of unique individuals.

- Only Swedish-born individuals born by Swedish parents were included. It therefore remains for a future study to show if the results may be generalized to other populations.

- The data did not include biological and environmental risk factors, to make up for the absence of such data we adjusted for educational level, which is related to lifestyle factors.

-Due to the cross-sectional design a temporal and possible causal association remains to be determined.

For peer review only

For peer review only - http://bmjopen.bmj.com/site/about/guidelines.xhtml

Introduction

Venous thromboembolism (VTE) is the third most common cardiovascular disease with an incidence of around 1-3 per 1000 individuals.[1-4] The incidence rate increases with age. VTE is rare among children with an incidence of approximately 1 per 100000 and common among elderly people >70 years and above with an incidence of around 1% per year.[5] The lifetime risk of VTE could be up to 10% in certain countries, for instance in Sweden.[3,4] VTE usually manifests as deep venous thrombosis (DVT) of the lower extremities or as pulmonary embolism (PE).[6] The consequences of VTE range from post-thrombotic syndrome, which affects approximately 20% of patients with DVT, to acute death in 1-2% of PE patients.[7,8] The 90-day mortality after VTE is around 5%.[9]

Risk factors for VTE are either acquired or genetic but they often interact.[8] Among the genetic risk factors, the most prominent are the classical thrombophilia, i.e. factor V Leiden (rs6025), the prothrombin G20210A mutation (rs1799963), deficiencies of antithrombin, protein C and protein S.[10,11] Among the acquired risk factors, age is the most significant with a steeply increased risk with advanced age.[5,6] Other acquired risk factors are for instance immobilization, trauma and surgery, malignancy, pregnancy and puerperium, oral contraception, and autoimmune disorders.[1,2,6,12,13]

Risk factors for VTE may work alone or in concert.[6,12] Multimorbidity, i.e. the presence of two or more diseases, increases with age.[14] As patients get older and the field of medicine advances the patients more often present with multiple diseases - multimorbidity.[14] However, multimorbidity also occurs among younger persons.[15] Genetic inheritance has been implicated in multimorbidity.[16] Dong et al have studied 439 common diseases among 385355 patients in

For peer review only - http://bmjopen.bmj.com/site/about/guidelines.xhtml

For peer review only

UK biobank. They identified 11285 multimorbidities and using genomewide association study (GWAS) data, they showed that 46% of multimorbidities were genetically interpretable (https://multimorbidity.comp-sysbio.org).[16] Due to this there is a need to investigate multimorbidity as a risk factor for additional illnesses, such as VTE which also have a genetic background. With the future demographic change with an increasingly elderly population with multimorbidity, there may be a need to adopt a multi-disease framework for clinical diagnostics and treatment. Despite the need to characterize multimorbidity there is no established definition of which morbidities should be included to define multimorbidity.[17] Multimorbidity has often been defined as two or more chronic non-communicable diseases (NCD).[17,18] Barnett et al used 40 diseases in a Scottish cross-sectional study and defined multimorbidity as the presence of two or more diseases where the prevalence of multimorbidity was 23.2% and was present in most people 65 years of age and older.[14] Although the frequency of multimorbidity increases with age, the burden of multimorbidity may be more common in those aged 65 years or younger, and this is especially common in socially deprived neighborhoods.[19] Several NCDs, but not all, included in the multimorbidity study by Barnett et al[14] have been associated with VTE.[20-39] For some associations between NCDs and VTE the associations are bidirectional,[36-38] e.g atherosclerotic disease and atrial fibrillation, but for most NCDs the association with VTE is unidirectional.[20-35] However, to the best of our knowledge, the association between multimorbidity and its severity (i.e. number of NCDs) and VTE has not been studied.

We aimed to determine whether there is an association between multimorbidity and its severity (i.e. number of NCDS) and VTE. We used large Swedish national registers to study the association between multimorbidity and VTE. A modified list of 45 NCDs diagnoses used by Barnett et al was used to define multimorbidity as the presence of two or more of 45

For peer review only - http://bmjopen.bmj.com/site/about/guidelines.xhtml

NCDs.[14] To determine a possible association between multimorbidity and VTE we also examined the association between the number of diseases and VTE. We also investigated whether a familial association of multimorbidity and VTE exists. Familial associations of a disease may be due to shared environmental exposure(s), shared genetic susceptibility, or a combination of the two [40] but familial association of a disease is a necessary, though insufficient, condition to infer the importance of genetic susceptibility.[40]

Material and methods

Registers

The following registers were used in the study: The Swedish Multi-Generation Register contains data of familial relationships; the National Patient Register (NPR) contains hospital discharge diagnoses between 1964-2015 with nationwide coverage from 1987 and from 2001-2015 outpatient hospital diagnoses; the Total Population Register with data on death date, education, marital status, migration; and the Swedish cause of death register.[41-46] We used the unique Swedish personal identity number (PIN) to link the databases. The PIN was, however, replaced with a serial number to ensure confidentiality by Statistics Sweden. The validity of Swedish registers has been investigated previously and they have a high degree of coverage and validity.[41-46] Statistics Sweden and the National Board of Health and Welfare provided the register data to us.

Study design

A nationwide extended cross-sectional study between 1997-2015. This period was chosen to increase the coverage of the registers and to use only the latest versions of the international

For peer review only - http://bmjopen.bmj.com/site/about/guidelines.xhtml

classification of disease (ICD) 10 codes. To minimize the risk of an incorrect PIN only Swedish-born individuals were included.[42] To investigate any possible familial aggregation we included pairs of full siblings born between 1948 and 2005 by Swedish parents that could further be linked to their Swedish-born half-siblings, and cousins to minimize this source of error. Parents in the study were born between 1932 and 1985 in Sweden. The Swedish National Multi-Generation Register was used to link relatives. We excluded the families with members who died or emigrated before 1997 or emigrated before the age of 17. Both biological parents were obligatorily known, i.e. pedigrees with two parents and two children. Full siblings were thereafter linked with related families and with the full siblings' half-siblings and cousins. Full siblings will hereafter be referred to as “siblings”. One dataset with all individuals entered only once was created. Four datasets for familial relations were created: twins, siblings, half-siblings, and cousins. In the four familial datasets, all relative pairs were double-entered, i.e. all sibling pairs, all twins’ pairs, all half-sibling pairs, and all cousin pairs, as described previously.[47] The double entry approach is a common procedure in genetics.[47,48] Zygosity was assessed only from sex, i.e. twins with different sex were considered to be DZ twins. We had no access to zygosity data. We allowed the same person to be included in more than one family relationship.

Definition of venous thromboembolism

Cases of VTE (i.e., affection status) were classified according to the World Health Organization International Classification of Diseases, Tenth Revision (ICD-10). VTE was defined using the hospital discharge register, main and supplementary diagnoses (between 1997 and 2015) by the following ICD codes: ICD-10: I80 (not I80.0) and I26.[11] It was also defined in the Outpatient Care Register (between 2001 and 2015) by the same ICD-10 codes as above and the cause of death register. Thus, VTE included pulmonary embolism and deep

For peer review only - http://bmjopen.bmj.com/site/about/guidelines.xhtml

vein thrombosis of the lower extremities. The diagnosis of VTE in the NPR has been shown to have an accuracy of 95%, whereas the overall validity of the NPR is 87%.[43,48] A quality control of the NPR register was performed for 118 patients with VTE in the Malmö diet and cancer study cohort. In 106 (90%) of 118 cases the diagnosis of VTE was correct.[4]

Multimorbidity score

Since there is no clear definition of which NCDs should be included, the NCDs used in Barnett et al. were used and modified to adapt to the Swedish ICD-10 codes (Supplementary Table S1). Further modifications were made to the list and infectious diseases were excluded. Five other clinically important and common NCDs contributing to disease burden were added instead: pancreatitis, obesity, gout, arthrosis, and osteoporosis. Furthermore, psoriasis and dermatitis or eczema were counted separately. These modifications were made to increase the number of NCDs to get a more comprehensive list. Thus, a total of 45 diagnoses were included and an individual with two or more NCDs was considered to have multimorbidity. Each diagnosis in an individual during the study period was awarded one point per disease and was cumulatively counted. An individual could theoretically have 45 points. In the study, no individual obtained more than 20 points.

Statistical analysis

Logistic regression was used to investigate the association between VTE and multimorbidity. Multimorbidity was defined as two or more diseases. Multimorbidity was also investigated as a cumulative score in order to determine any possible increasing association. Results are presented as odds ratios (ORs) with 95% confidence intervals (95%CI). Adjustments were made for year of birth, sex, region at birth and level of education. Stratified analysis was performed according to sex and birth year quartiles. The familial associations were

For peer review only - http://bmjopen.bmj.com/site/about/guidelines.xhtml

determined using the double-entry approach.[47] The ambiguity in unselected samples as to which twin's, sibling's, half-sibling's or cousin's trait should be used as the dependent, and which as the independent variable, could be resolved by using double-entry.[47] Each twin, sibling, half-sibling, or cousin is entered twice in the data, and each member of a twin, sibling, half-sibling, or cousin pair provides once the dependent and once the explanatory variable.[47] While the consistency of the regression estimates for heritability and environmental influences is not affected by double-entry, the standard errors of the coefficients are biased and need to be adjusted.[47] In the present study in the logistic regression models, we used the "variance covariance (vce) cluster" method using STATA, which calculated robust standard errors using families as clusters.[49] The vce (cluster clustvar) specifies that the standard errors allow for intragroup correlation, relaxing the usual requirement that the observations be independent.

Statistical significance was set to $p<0,05$, all tests were 2-tailed. Data were analyzed using STATA version 16.1 (StataCorp LLC, College Station, TX, USA)

Patient and Public Involvement

None.

For peer review only

For peer review only - http://bmjopen.bmj.com/site/about/guidelines.xhtml

Results

Multimorbidity

A total of 2,694,442 unique individuals were included in the study, 49% of these were females. The mean age at the end of the study was 32 years. During the study period between 1997 and 2015, a total of 440,742 (16.36%) individuals were diagnosed with multimorbidity (Table 1). Of these 253,931 (57.61%) were females and the proportion of females increased with increasing multimorbidity score with 63.27% of the individuals with a score of ≥5 being female (Supplementary Table 2). Multimorbidity was associated with level of education where 25.47% of the multimorbid individuals had an education of ≥12 years compared to 31.32% in the non-multimorbid group (Table 1). Multimorbidity also increased with older age at the end of the study, multimorbid individuals had a mean age of 35 years versus 31 in non-multimorbid groups. Baseline characteristics can be found in Table 1 and in Supplementary Table 2.

Table 1. Descriptive findings for all unique study participants with stratification according to multimorbidity scores for number of unique individuals, sex, education, age at the end of study, birth date, and VTE (venous thromboembolism).

		*Multimorbidity scores	
	All	≤ 1	≥ 2
Unique individuals, % (n)	100% (n=2694442)	83.64% (n=2253700)	16.36% (n=440742)
Sex, Female, % (n)	48.73% (n=1312989)	80.66% (n=1059058)	19.34% (n=253931)
Education (≥12 years), % (n)	30.36% (n=818146)	86.28% (n=705901)	13.72% (n=112245)
Age at end of study (years),<br>Median (IQR)<br>Range (min-max)	32<br>(22-43)<br>(0 -68)	31<br>(22-42)<br>(0-67)	35<br>(24-46)<br>(0-68)
Year of birth, Median (IQR)<br>Range (min-max)	1983<br>(1972-1993)<br>(1947-2005)	1984<br>(1973-1993)<br>(1948-2005)	1980<br>(1969-1990)<br>(1947-2005)
VTE, % (n)	0.6%<br>(n=16099)	56.62%<br>(n=9116)	43.38%<br>(n=6983)

IQR=Interquartile Range. n=number of individuals. *%=For multimorbidity scores percentage is calculated from the total number of people given in the first colum (All column).

For peer review only - http://bmjopen.bmj.com/site/about/guidelines.xhtml

VTE

There was a total of 16,099 cases with diagnosed VTE during the study period between 1997 and 2015. In individuals with multimorbidity, 1.58 % had VTE compared to 0.40% in individuals without multimorbidity. Of the female individuals 0.66% had VTE and among males the figure was 0.54%. VTE increased with age with 1.3% in the oldest group and 0.08% in the youngest group. (Tables 1-3).

Table 2. Odds ratio (OR) for VTE (venous thromboembolism) and multimorbidity (score ≥2) for all and stratified according to sex. Odds Ratios (ORs) with 95 % confidence interval (CI) for multimorbidity. Reference with one or no diseases (score=0 or 1). Total observations n=2694442.

	OR (95% CI)			
	No VTE	VTE	Model 1	Model 2
All	433759	6983	3.96 (3.84-4.09)	3.16 (3.06-3.27)
Males	183732	3079	4.52 (4.32-4.74)	3.45 (3.29-3.62)
Females	250027	3904	3.5 (3.35-3.65)	2.91 (2.79-3.04)

Abbreviations: OR, odds ratio. Model 1 is a crude model (univariable). Model 2 is an adjusted model (multivariable), with adjustments for year of birth, county, and educational attainment.

Table 3. Odds ratio for VTE (venous thromboembolism) according to multimorbidity score (0 to ≥2) and age stratified according to interquartile range (IQR1-4). Odds Ratios (ORs) with 95 % confidence interval (CI) for multimorbidity scores. Reference with no diseases (score=0). Total observations n=2694442.

	OR (95% CI)			
Age groups	No VTE	VTE	Model 1	Model 2
IQR1	135989	4032	3.35 (3.21-3.50)	3.14 (3.01-3.29)
IQR2	106001	1857	3.43 (3.24-3.64)	3.14 (2.96-3.34)
IQR3	104897	919	3.48 (3.21-3.78)	3.09 (2.84-3.37)
IQR4	86872	175	3.52 (2.93-4.22)	3.07 (2.56-3.7)

Abbreviations: OR, odds ratio. Model 1 is a crude model (univariable). Model 2 is an adjusted model (multivariable), with adjustments for sex, year of birth, county, and educational attainment. IQR=interquartile range. IQR1 1947-1972, IQR2 1972-1983, IQR3 1983-1993, IQR4 1993-2005.

For peer review only - http://bmjopen.bmj.com/site/about/guidelines.xhtml

VTE and multimorbidity

In the multimorbidity group a total of 6983 of the 440742 (1.58%) individuals were diagnosed with VTE (Table 1). In the group with a multimorbidity score of ≥5 1603 of the 36313 (4.41%) had VTE during the study period (Supplementary Table 2). The OR for VTE in the adjusted model for an individual with multimorbidity was 3.16 (95%CI 3.06 to 3.27) (Table 2). For males with multimorbidity, the OR for VTE was higher than for females (Table 2). Birth year was stratified into quartiles. For individuals born 1947-1972 the OR for VTE with multimorbidity was 3.14 (95%CI: 3.01 to 3.29) in the adjusted model and for individuals born 1993-2005 the OR for VTE with multimorbidity was 3.07 (95%CI 2.56 to 3.7) (Table 3). Thus, there was no strong age dependence regarding the association between multimorbidity and VTE.

Association between increasing multimorbidity score and VTE

The association of increasing multimorbidity score on VTE was investigated (Table 4). It showed an increased OR for increasing score of multimorbidity. OR for a multimorbidity score of two in the adjusted model was 2.93 (95%CI 2.80 to 3.08), 4.07 (95%CI 3.85 to 4.31) for a multimorbidity score of three, 5.46 (95%CI: 5.10 to 5.85) for a multimorbidity score of four, and for a multimorbidity score of five or more the OR was 9.08 (95%CI: 8.56 to 9.64). (Table 4).

Table 4. Odds ratio for VTE (venous thromboembolism) according to multimorbidity score (0 to ≥5). Odds Ratios (ORs) with 95 % confidence interval (CI) for multimorbidity scores. Reference with no diseases (score=0).

	All (n=2694442)			
	OR (95% CI)			
Multimorbidity score	No VTE	VTE	Model 1	Model 2
Score 0	1611205	5008	1[Reference]	1[Reference]

For peer review only - http://bmjopen.bmj.com/site/about/guidelines.xhtml

Score 1	633379	4108	2.09 (2.00-2.18)	1.94 (1.86-2.02)
Score 2	251558	2652	3.39 (3.24-3.56)	2.93 (2.80-3.08)
Score 3	102969	1665	5.20 (4.92-5.50)	4.07 (3.85-4.31)
Score 4	44522	1063	7.68 (7.18-8.21)	5.46(5.10-5.85)
Score ≥5	34710	1603	14.86 (14.03-15.73)	9.08 (8.56-9.64)

Abbreviations: OR, odds ratio. Model 1 is a crude model (univariable). Model 2 is an adjusted model (multivariable), with adjustments for sex, year of birth, county, and educational attainment.

Age and sex stratification

Multimorbidity score results for sex and age stratification are presented in Supplementary Tables S3-S8. A strong association between multimorbidity and VTE could be observed in both sexes and all age groups.

Familial associations between multimorbidity and VTE

There was mostly a weak association between twin, sibling, half-sibling and cousin history of multimorbidity and VTE (Table 5). The ORs in adjusted models for VTE in patients with multimorbidity were 1.89 in twins (95%CI 1.17 to 3.05), 1.18 in siblings (95%CI 1.12 to

For peer review only - http://bmjopen.bmj.com/site/about/guidelines.xhtml

For peer review only

1.24), 1.1 in half-siblings (95%CI 1.03 to 1.18), and 1.07 in cousins (95%CI 1.04 to 1.1) of those diagnosed with index score more than one (Table 5). Among twins, the adjusted OR for VTE in patients with multimorbidity was =0.73 (95%CI 0.04 to 1.35) for twin score=1, OR=1.57 (95%CI 0.81 to 3.05) for twin score=2, OR=1.26 (95%CI 0.48 to 3.31) for twin score=3, OR=1.48 (95%CI 0.44 to 4.98) for twin score=4, and OR=3.98 (95%CI 1.17 to 9.39) for twin score ≥ 5.

For peer review

Table 5. Familial associations for multimorbidity and venous thromboembolism (VTE). Odds ratio with 95 % confidence interval (CI) for VTE according to multimorbidity score (0 to ≥5) or multimorbidity (≥ 2 score) in relatives. Reference are individuals with relatives with no diseases (score=0) for the graded score (score between 1 and ≥5). For multimorbidity (score≥2) reference are individuals with relatives with no multimorbidity (score<2), i.e. no or one disease. Zygosity of twins are not known.

	Twins (n=24020)		Siblings (n=1546108)		Half-siblings (n=984976)		Cousins (n=6623156)	
	OR (95% CI)		OR (95% CI)		OR (95% CI)		OR (95% CI)	
VTE risk in individuals with relatives with score between 1 and ≥5.								
Relative score	Model 1	Model 2	Model 1	Model 2	Model 1	Model 2	Model 1	Model 2
Score 0	Reference	Reference	Reference	Reference	Reference	Reference	Reference	Reference

Score 1	0.77 (0.42-1.41)	0.73 (0.4-1.35)	1.19 (1.13-1.25)	1.11 (1.06-1.17)	1.12 (1.05-1.19)	1.08 (1.01-1.15)	1.09(1.06-1.12)	1.04(1.02-1.07)
Score 2	1.81 (0.95-3.46)	1.57 (0.81-3.05)	1.37 (1.28-1.46)	1.19 (1.11-1.27)	1.14 (1.05-1.25)	1.07 (0.98-1.17)	1.16(1.11-1.2)	1.07(1.03-1.11)
Score 3	1.86 (0.74-4.68)	1.26 (0.48-3.31)	1.5 (1.37-1.67)	1.18 (1.07-1.3)	1.35 (1.2-1.51)	1.19 (1.07-1.34)	1.21(1.14-1.27)	1.06(1-1.11)
Score 4	2.66 (0.82-8.59)	1.48 (0.44-4.98)	1.89 (1.67-2.15)	1.34 (1.18-1.53)	1.4 (1.19-1.64)	1.17 (1.0-1.37)	1.38(1.28-1.48)	1.14(1.06-1.22)
Score ≥5	9.31 (4.17-20.79)	3.98 (1.69-9.39)	2.16 (1.88-2.48)	1.31 (1.14-1.51)	1.6 (1.36-1.89)	1.2 (1.02-1.41)	1.52(1.41-1.65)	1.16(1.07-1.26)
VTE risk in individuals with relatives with multimorbidity (Score ≥2).								
Score <2	Reference	Reference	Reference	Reference	Reference	Reference	Reference	Reference
Score ≥2	2.55 (1.62-4.02)	1.89 (1.17-3.05)	1.43 (1.36-1.51)	1.18 (1.12-1.24)	1.23 (1.15-1.31)	1.1 (1.03-1.18)	1.19(1.16-1.23)	1.07(1.04-1.1)
Abbreviations: OR=odds ratio. The ORs were derived from double entry and the variance covariance (vce) cluster method was used. Model 1 is a crude model. Model 2 is adjusted for sex, year of birth, county, and educational attainment.								

For peer review only - http://bmjopen.bmj.com/site/about/guidelines.xhtml

Discussion

The strong association between multimorbidity and VTE is, to the best of our knowledge, a novel finding. However, the study design does not allow any causal inferences to be drawn regarding multimorbidity and VTE. Still, the strong and increasing association with increasing multimorbidity score suggests that future cohort studies might be considered in order to determine whether a possible causal association between multimorbidity and VTE exists.[50] Our present study shows an association between multimorbidity and VTE with an adjusted OR of 3.16. The VTE risk increases with a higher multimorbidity score and is nine times higher in individuals with a multimorbidity score of five or more compared to individuals with no morbidity. Thus, multimorbidity is strongly associated with VTE. Genetic inheritance has been implicated in multimorbidity.[16] However, our data argue against a major genetic association between multimorbidity and VTE due to the mostly weak familial associations. However, there was a significant familial association, which correlated with the degree of relatedness suggesting a possible shared genetic susceptibility to the association between multimorbidity and VTE, though shared environmental exposures also may be involved. A high multimorbidity score in a patient confers similar odds ratios for VTE as strong genetic or acquired risk factors.[1,2,6,10-12] Moroever, multimorbidity has been associated with inflammatory biomarkers and an association between inflammation and VTE has long been recognized.[51-54] Based on these findings, future dissection of multimorbidity and its relation to VTE in cohort studies could be of value.

The impact of multimorbidity on VTE is stronger in males compared to females and with a higher relationship among males. On the other hand, females have increased multimorbidity and live longer which may give a double burden for the female population in the context risk of VTE, if multimorbidity will turn out to be an important risk factor for VTE.

For peer review only - http://bmjopen.bmj.com/site/about/guidelines.xhtml

The risk of multimorbidity is increasing with age and more individuals worldwide are multimorbid.[16] Our study population is relatively young and confirms previous studies that multimorbidity may also affect young persons.[15] However, there was a strong association with multimorbidity in all age groups suggesting that multimorbidity might also be of importance in older age groups not studied here; this needs to be confirmed in further studies. If multimorbidity in future studies turn out to be a risk factor for VTE, it is possible that multimorbidity is an important contributor to the high incidence of VTE in society due to its high prevalence. Common risk factors, both genetic and acquired, are often seen as individual risk factors in specific settings, like surgery, pregnancy, puerperium, oral contraceptives, surgery and trauma as well as for specific diseases, such as cancer. The present study suggests that we might count the total burden of disease into a multimorbidity score to get a better risk estimate of VTE. However, future prospective studies need to determine which combinations of diseases are most strongly associated with VTE.

Since there is no clear definition of which diagnoses should be used in the field of multimorbidity the ones that are included are sometimes random.[14-19] In this study we used a modified score validated by Barnett et al. 2012.[14] The NCDs that were used in this study are all common. They all have an impact on healthcare, and most are considerably stable during an individual's life and can coexist and create a significant health burden.[14] It will be important to investigate whether a certain combination of diagnoses and multimorbidity has an especially strong association with VTE and further studies are needed to investigate this matter. However, the association between multimorbidity and VTE suggests that not only specific diseases are important but also the total burden of certain diseases.

For peer review only - http://bmjopen.bmj.com/site/about/guidelines.xhtml

Strengths and limitations

The limited number of confounders is a potential limitation. An important limitation of the study is that we did not determine any temporal association between multimorbidity and VTE. We therefore cannot draw any causal inferences or exclude reverse causality. However, only atrial fibrillation and arterial atherosclerotic diseases have been described to exhibit a bidirectional association with VTE.[36-38] Moreover, it is possible that certain combinations of diseases are more strongly associated with VTE than others. It will be of importance to further dissect the occurrence of multimorbidity and its relation to future risk for VTE in cohort studies. The study period was chosen to minimize the risk of missed data and to increase the coverage of individuals. Furthermore, having more comprehensive ICD-10 codes decreases the risk of inaccurate coding from the registers. A strength of the study is the high coverage and high validity of the registers and the large number of unique individuals.[41-46] A limitation is that only Swedish-born individuals born by Swedish parents were included. It therefore remains for a future study to show if the results may be generalized to other populations. Another limitation is that the data did not include biological and environmental risk factors such as smoking, alcohol consumption, height, weight, blood pressure and exercise habits. To make up for the absence of such data we adjusted for educational level, which is related to lifestyle factors.[55] Also, the included patients were relatively young with an age-range of 0-68 and a mean of 32 at the end of the study and generalization of the elderly is uncertain, even though within this age span between 0-68 years there was no age effect on the association. Hence there is a need for further studies in the future.

For peer review only - http://bmjopen.bmj.com/site/about/guidelines.xhtml

Conclusion

We have shown that an association exists between multimorbidity and multimorbidity severity (i.e. number of NCDs) and venous thromboembolism. Weak shared familial factors (environmental or genetic) may also be involved. The relation between multimorbidity and VTE could be further studied in cohort studies and Mendelian randomization studies in order to determine a temporal and possibly causal association.

Contributorship statement

JA, MP, BH, JS, AH, KS and BZ contributed to the conception and design of the study; JS and KS contributed to the acquisition of data; JA, MP, BH, JS, AH, KS and BZ contributed to the analysis and interpretation of data; JA drafted the manuscript; and JA, MP, BH, JS, AH, KS and BZ revised it critically and approved the final version. JA, MP, BH, JS, AH, KS and BZ had full access to all the data (including statistical reports and tables) and take responsibility for the integrity of the data and the accuracy of their analysis.

Competing interests

The authors have nothing to disclose.

Funding

ALF-funding from Region Skåne (N/A) and the Swedish Research Council (2020-01824).

Data sharing statement

We are not allowed to share our data as the data are drawn from registries owned by a third party, i.e. the Swedish authorities. The reason behind this is that public availability could potentially compromise patient confidentiality or privacy. Permission to use the data is issued

For peer review only

For peer review only - http://bmjopen.bmj.com/site/about/guidelines.xhtml

to researchers (if certain conditions are fulfilled) by the National Board of Health and Welfare and by Statistics Sweden. Request to use the data can be applied for from the Swedish National Board of Health and Welfare (https://www.socialstyrelsen.se/en/statistics-and-data/statistics/statistical-database/) and Statistics Sweden (https://www.scb.se/en/About-us/contact-us/). An application for use of the same minimal dataset, which we have used in this study, should include a motivation, a referral to the present study and an addition of a list of variables, before submission of a data request to the Swedish National Board of Health and Welfare and Statistics Sweden using the contact addresses above. Then the authorities will coordinate the handling of the request and do a special review.

Ethics statement

This study was approved by the Regional Ethical Review Board in Lund (2012/795 and later amendments).

Acknowledgments

We thank Patrick Reilly, science editor at Center for Primary Health Care Research, Department of Clinical Science, Lund University and Region Skåne, Malmö, Sweden, for language review.

References

1. White RH. The epidemiology of venous thromboembolism. Circulation. 2003;107(23 Suppl 1):I4-8.
2. Heit JA. Epidemiology of venous thromboembolism. Nat Rev Cardiol. 2015;12:464-74.
3. Hansson PO, Welin L, Tibblin G, Eriksson H. Deep vein thrombosis and pulmonary embolism in the general population. 'The Study of Men Born in 1913'. Arch Intern Med. 1997;157(15):1665-70.
4. Manderstedt E, Halldén C, Lind-Halldén C, Elf J, Svensson PJ, Engström G, Melander O, Baras A, Lotta LA, Zöller B; Regeneron Genetics Center. Thrombomodulin (THBD) gene variants and thrombotic risk in a population-based cohort study. J Thromb Haemost. 2022;20(4):929-935.

For peer review only - http://bmjopen.bmj.com/site/about/guidelines.xhtml

For peer review only

5. Naess IA, Christiansen SC, Romundstad P, Cannegieter SC, Rosendaal FR, Hammerstrøm J. Incidence and mortality of venous thrombosis: a population-based study. J Thromb Haemost. 2007;5(4):692-9.
6. Rosendaal FR. Venous thrombosis: a multicausal disease. Lancet. 1999;353(9159):1167-73.
7. Brandjes DP, Büller HR, Heijboer H, Huisman MV, de Rijk M, Jagt H, ten Cate JW. Randomised trial of effect of compression stockings in patients with symptomatic proximal-vein thrombosis. Lancet. 1997;349(9054):759-62.
8. Anderson FA Jr, Wheeler HB, Goldberg RJ, Hosmer DW, Patwardhan NA, Jovanovic B, Forcier A, Dalen JE. A population-based perspective of the hospital incidence and case-fatality rates of deep vein thrombosis and pulmonary embolism. The Worcester DVT Study. Arch Intern Med. 1991;151(5):933-8.
9. Zöller B, Pirouzifard M, Sundquist J, Sundquist K. Association of Short-Term Mortality of Venous Thromboembolism with Family History of Venous Thromboembolism and Charlson Comorbidity Index. Thromb Haemost. 2019;119(1):48-55.
10. Zöller B, Svensson PJ, Dahlbäck B, Lind-Hallden C, Hallden C, Elf J. Genetic risk factors for venous thromboembolism. Expert Rev Hematol. 2020;13(9):971-981.
11. Manderstedt E, Manderstedt E, Lind-Halldén C, Halldén C, Elf J, Svensson PJ, Dahlbäck B, Engström G, Melander O, Baras A, Lotta LA, Zöller B; Regeneron Genetics Center 4 [Link]. Classic Thrombophilias and Thrombotic Risk Among Middle-Aged and Older Adults: A Population-Based Cohort Study. J Am Heart Assoc. 2022;11(4):e023018.
12. Lijfering WM, Rosendaal FR, Cannegieter SC. Risk factors for venous thrombosis - current understanding from an epidemiological point of view. Br J Haematol. 2010;149(6):824-33.
13. Zöller B, Li X, Sundquist J, Sundquist K. Risk of pulmonary embolism in patients with autoimmune disorders: a nationwide follow-up study from Sweden. Lancet. 2012;379(9812):244-9.
14. Barnett K, Mercer SW, Norbury M, Watt G, Wyke S, Guthrie B. Epidemiology of multimorbidity and implications for health care, research, and medical education: a cross-sectional study. Lancet. 2012;380(9836):37-43.
15. Troelstra SA, Straker L, Harris M, Brown S, van der Beek AJ, Coenen P. Multimorbidity is common among young workers and related to increased work absenteeism and presenteeism: results from the population-based Raine Study cohort. Scand J Work Environ Health. 2020;46(2):218-227.
16. Dong G, Feng J, Sun F, Chen J, Zhao XM. A global overview of genetically interpretable multimorbidities among common diseases in the UK Biobank. Genome Med. 2021;13(1):110.
17. Johnston MC, Crilly M, Black C, Prescott GJ, Mercer SW. Defining and measuring multimorbidity: a systematic review of systematic reviews. Eur J Public Health. 2019 Feb 1;29(1):182-189.
18. Pearson-Stuttard J, Ezzati M, Gregg EW. Multimorbidity-a defining challenge for health systems. Lancet Public Health. 2019;4(12):e599-e600.
19. Wallace E, Salisbury C, Guthrie B, Lewis C, Fahey T, Smith SM. Managing patients with multimorbidity in primary care. BMJ. 2015;350:h176.

For peer review only - http://bmjopen.bmj.com/site/about/guidelines.xhtml

20. Wang Y, Ding C, Guo C, Wang J, Liu S. Association between thyroid dysfunction and venous thromboembolism: A systematic review and meta-analysis. Medicine (Baltimore). 2023;102(11):e33301.
21. Guo Y, Zhou F, Xu H. Gout and risk of venous thromboembolism: A systematic review and meta-analysis of cohort studies. Int J Rheum Dis. 2023;26(2):344-353.
22. Allman-Farinelli MA. Obesity and venous thrombosis: a review. Semin Thromb Hemost. 2011;37(8):903-7.
23. Zhang XY, Dong HC, Wang WF, Zhang Y. Risk of venous thromboembolism in children and adolescents with inflammatory bowel disease: A systematic review and meta-analysis. World J Gastroenterol. 2022;28(16):1705-1717.
24. Kowal C, Peyre H, Amad A, Pelissolo A, Leboyer M, Schürhoff F, Pignon B. Psychotic, Mood, and Anxiety Disorders and Venous Thromboembolism: A Systematic Review and Meta-Analysis. Psychosom Med. 2020;82(9):838-849.
25. Tang L, Wu YY, Lip GY, Yin P, Hu Y. Heart failure and risk of venous thromboembolism: a systematic review and meta-analysis. Lancet Haematol. 2016;3(1):e30-44.
26. Bai J, Ding X, Du X, Zhao X, Wang Z, Ma Z. Diabetes is associated with increased risk of venous thromboembolism: a systematic review and meta-analysis. Thromb Res. 2015;135(1):90-5.
27. Gariani K, Mavrakanas T, Combescure C, Perrier A, Marti C. Is diabetes mellitus a risk factor for venous thromboembolism? A systematic review and meta-analysis of case-control and cohort studies. Eur J Intern Med. 2016;28:52-8.
28. Bell EJ, Folsom AR, Lutsey PL, Selvin E, Zakai NA, Cushman M, Alonso A. Diabetes mellitus and venous thromboembolism: A systematic review and meta-analysis. Diabetes Res Clin Pract. 2016;111:10-8.
29. Ahmed O, Geraldes R, DeLuca GC, Palace J. Multiple sclerosis and the risk of systemic venous thrombosis: A systematic review. Mult Scler Relat Disord. 2019;27:424-430.
30. Ungprasert P, Sanguankeo A, Upala S, Suksaranjit P. Psoriasis and risk of venous thromboembolism: a systematic review and meta-analysis. QJM. 2014;107(10):793-7.
31. Horsted F, West J, Grainge MJ. Risk of venous thromboembolism in patients with cancer: a systematic review and meta-analysis. PLoS Med. 2012;9(7):e1001275.
32. Qi X, Ren W, Guo X, Fan D. Epidemiology of venous thromboembolism in patients with liver diseases: a systematic review and meta-analysis. Intern Emerg Med. 2015;10(2):205-17.
33. Hu LJ, Ji B, Fan HX. Venous thromboembolism risk in rheumatoid arthritis patients: a systematic review and updated meta-analysis. Eur Rev Med Pharmacol Sci. 2021;25(22):7005-7013.
34. Bello N, Meyers KJ, Workman J, Marcano Belisario J, Cervera R. Systematic Literature Review and Meta-analysis of Venous Thromboembolism Events in Systemic Lupus Erythematosus. Rheumatol Ther. 2023;10(1):7-34.
35. Zöller B, Pirouzifard M, Memon AA, Sundquist J, Sundquist K. Risk of pulmonary embolism and deep venous thrombosis in patients with asthma: a nationwide case-control study from Sweden. Eur Respir J. 2017;49(2):1601014
36. Bikdeli B, Abou Ziki MD, Lip GYH. Pulmonary Embolism and Atrial Fibrillation: Two Sides of the Same Coin? A Systematic Review. Semin Thromb Hemost. 2017;43(8):849-863.

For peer review only - http://bmjopen.bmj.com/site/about/guidelines.xhtml

37. Prandoni P, Bilora F, Marchiori A, Bernardi E, Petrobelli F, Lensing AW, Prins MH, Girolami A. An association between atherosclerosis and venous thrombosis. N Engl J Med. 2003;348(15):1435-41.
38. Becattini C, Vedovati MC, Ageno W, Dentali F, Agnelli G. Incidence of arterial cardiovascular events after venous thromboembolism: a systematic review and a meta-analysis. J Thromb Haemost. 2010;8(5):891-7.
39. Chen TL, Lee LL, Huang HK, Chen LY, Loh CH, Chi CC. Association of Risk of Incident Venous Thromboembolism With Atopic Dermatitis and Treatment With Janus Kinase Inhibitors: A Systematic Review and Meta-analysis. JAMA Dermatol. 2022;158(11):1254-1261.
40. Risch N. The genetic epidemiology of cancer: interpreting family and twin studies and their implications for molecular genetic approaches. Cancer Epidemiol Biomarkers Prev. 2001;10:733-41
41. Rosen M, Hakulinen T. Use of disease registers. In: Ahrens W, Pigeot I, eds. Handbook of Epidemiology. Springer-Verlag; 2005:231–252.
42. Ludvigsson JF, Otterblad-Olausson P, Pettersson BU, Ekbom A. The Swedish personal identity number: possibilities and pitfalls in healthcare and medical research. Eur J Epidemiol. 2009;24(11):659-67.
43. Ludvigsson JF, Andersson E, Ekbom A, Feychting M, Kim JL, Reuterwall C, Heurgren M, Olausson PO. External review and validation of the Swedish national inpatient register. BMC Public Health. 2011;11:450.
44. Ludvigsson JF, Almqvist C, Bonamy AK, Ljung R, Michaëlsson K, Neovius M, Stephansson O, Ye W. Registers of the Swedish total population and their use in medical research. Eur J Epidemiol. 2016;31(2):125-36.
45. Ekbom A. The Swedish Multi-generation Register. Methods Mol Biol. 2011;675:215-20.
46. Zöller B. Nationwide family studies of cardiovascular diseases—clinical and genetic implications of family history. EMJ Cardiol. 2013;1:102–113.
47. Kohler HP, Rodgers JL. DF-analyses of heritability with double-entry twin data: asymptotic standard errors and efficient estimation. Behav Genet. 2001 Mar;31(2):179-91.
48. Rosengren A, Fredén M, Hansson PO, Wilhelmsen L, Wedel H, Eriksson H. Psychosocial factors and venous thromboembolism: a long-term follow-up study of Swedish men. J Thromb Haemost. 2008;6(4):558-64.
49. McLoughlin G, Palmer JA, Rijsdijk F, Makeig S. Genetic overlap between evoked frontocentral theta-band phase variability, reaction time variability, and attention-deficit/hyperactivity disorder symptoms in a twin study. Biol Psychiatry. 2014; 75: 238-47.
50. Hill AB. The environment and disease: association or causation? Proc R Soc Med. 1965;58(5):295-300.
51. St Sauver J, Rocca W, LeBrasseur N, Chamberlain A, Olson J, Jacobson D, McGree M, Mielke M. Inflammatory biomarkers, multi-morbidity, and biologic aging. J Int Med Res. 2022;50(7):3000605221109393.
52. Fabbri E, An Y, Zoli M, Simonsick EM, Guralnik JM, Bandinelli S, Boyd CM, Ferrucci L. Aging and the burden of multimorbidity: associations with inflammatory and anabolic hormonal biomarkers. J Gerontol A Biol Sci Med Sci. 2015;70(1):63-70.

For peer review only - http://bmjopen.bmj.com/site/about/guidelines.xhtml

53. Wessman T, Tofik R, Ruge T, Melander O. Associations between biomarkers of multimorbidity burden and mortality risk among patients with acute dyspnea. Intern Emerg Med. 2022;17(2):559-567.
54. Colling ME, Tourdot BE, Kanthi Y. Inflammation, Infection and Venous Thromboembolism. Circ Res. 2021;128(12):2017-2036
55. Winkleby MA, Jatulis DE, Frank E, Fortmann SP. Socioeconomic status and health: how education, income, and occupation contribute to risk factors for cardiovascular disease. Am J Public Health. 1992;82(6):816-20.

For peer review only

For peer review only - http://bmjopen.bmj.com/site/about/guidelines.xhtml

Supplementary Appendix: Authors: Ahrén J, Pirouzifard M, Holmquist B, Sundquist J, Halling A, Sundquist K, Zöller B

Table of content pages 1-9 **Pages**

1. Supplementary Table S1 2-3
2. Supplementary Table S2 4
3. Supplementary Table S3 5
4. Supplementary Table S4 6
5. Supplementary Table S5 7
6. Supplementary Table S6 8
7. Supplementary Table S7 9
8. Supplementary Table S8 10

For peer review only

Supplementary Table S1. Diseases with ICD codes included in the multimorbidity score modified and adapted to Swedish ICD-10 codes after Barnett K et al (2012)*. Infectious diseases, i.e. viral hepatitis, were excluded and six common non-communicable diseases (NCDs) were added: dermatitis or eczema, gout, arthrosis, osteoporosis, obesity, and pancreatitis. One point for each disease. The theoretical range of the score is 0-45 points.

	Included disorders in the multimorbidity score	ICD-10**
1	Hypertension	I10-I15
2	Affective disorders	F30-F39
3	Painful back condition	M50-54
4	Asthma	J45-J46
5	CHD	I20-I25
6	Ulcer disease	K221, K25-K28
7	Diabetes	E10-E14
8	Thyroid disorders	E00-E07
9	Connective tissue disease	M05, M06, M08, M09, M30-M36, D86
10	Hearing loss (or impaired)	H90-H91
11	Chronic obstructive pulmonary disease (COPD)	J41-J44
12	Anxiety	F40-F48
13	Irritable bowel syndrome (IBS)	K58
14	Cancer	C00-C99
15	Alcohol use disorders	F10
16	Psychoactive substance misuse	F11-F14, F16, F18, F19
17	Constipation	K590
18	Cerebrovascular disease	I60-I69
19	Renal disease	N00-N19, Q61
20	Diverticular disease of intestine	K57
21	Atrial fibrillation	I48
22	Atherosclerosis	I70-I74, I77
23	Heart failure	I50, I110, I130, I132
24	Prostate disease	N40-N42
25	Glaucoma	H40, H42

For peer review only

26	Epilepsy	G40, G41
27	Dementia	F00-F03
28	Schizophrenia disorders	F20, F21
29	Psoriasis	L40
30	Dermatitis or Eczema	L20-L30
31	Inflammatory bowel disease (IBD)	K50, K51
32	Migraine	G43, G440, G441
33	Blindness & low vision	H53-H54
34	Chronic sinusitis	J32
35	Learning disability	F81
36	Anorexia or bulimia	F50
37	Bronchiectasis	J47
38	Parkinson's disease	G20, G21, G22
39	Multiple sclerosis	G35
40	Liver disease	K70-K77
41	Gout	M10
42	Arthrosis	M15-M19
43	Osteoporosis	M80-M82
44	Obesity	E65, E66
45	Pancreas diseases	K85, K86

*Barnett K, Mercer SW, Norbury M, Watt G, Wyke S, Guthrie B. Epidemiology of multimorbidity and implications for health care, research, and medical education: a cross-sectional study. Lancet 2012; 380: 37-43.

**ICD=International classification of disease.

For peer review only

Supplementary Table 2. Descriptive findings for all unique study participants with stratification according to multimorbidity scores for number of unique individuals, sex, education, age at the end of study, birth date, and VTE (venous thromboembolism).

		Multimorbidity scores							
	All	0	1	2	3	4	≥ 5	≤ 1	≥ 2
Unique individuals, n (%)	2694442 (100)	1616213 (59.98)	637487 (23.66)	254210 (9.43)	104634 (3.88)	45585 (1.69)	36313 (1.35)	2253700 (83.64)	440742 (16.36)
Sex, Female, % (n)	48.73 (1312989)	45.25 (731337)	51.41 (327721)	55.66 (141488)	59.07 (61808)	60.68 (27661)	63.27 (22974)	46.99 (1059058)	57.61 (253931)
Education (≥12 years), % (n)	30.36 (818146)	31.97 (516774)	29.67 (189127)	27.16 (69050)	24.80 (25946)	22.45 (10232)	19.32 (7017)	31.32 (705901)	25.47 (112245)
Age at end of study, Median (IQR) Range (min-max)	32 (22-43) (0 -68)	31 (22-42) (0-67)	32 (22-43) (0-67)	33 (23-45) (0-67)	36 (25-46) (1-66)	38 (27-48) (1-67)	42 (31-50) (3-68)	31 (22-42) (0-67)	35 (24-46) (0-68)
Year of birth, Median (IQR) Range (min-max)	1983 (1972-1993) (1947-2005)	1984 (1973-1993) (1948-2005)	1983 (1972-1993) (1948-2005)	1981 (1970-1992) (1948-2005)	1979 (1968-1990) (1949-2005)	1977 (1967-1988) (1948-2005)	1972 (1964-1984) (1947-2005)	1984 (1973-1993) (1948-2005)	1980 (1969-1990) (1947-2005)
VTE, n (%)	16099 (0.6)	5008 (0.31)	4108 (0.64)	2652 (1.04)	1665 (1.59)	1063 (2.33)	1603 (4.41)	9116 (0.40)	6983 (1.58)

IQR=Interquartile Range.

Supplementary Table S3. Odds ratio for VTE according to multimorbidity score (0 to ≥5) in males. Odds Ratios (ORs) with 95 % confidence interval (CI) for multimorbidity scores. Reference with no diseases (score=0).				
	Male (n=1381453)			
	OR (95% CI)			
VTE score	No VTE	VTE	Model 1	Model 2
Score 0	882367	2509	1[Reference]	1[Reference]
Score 1	307866	1900	2.17 (2.04-2.30)	2.03 (1.91-2.26)
Score 2	111533	1189	3.75 (3.5-4.02)	3.20 (2.99-3.44)
Score 3	42063	763	6.38 (5.88-6.92)	4.73 (4.35-5.14)
Score 4	17441	483	9.74 (8.83-10.75)	6.3(5.69-6.96)
Score ≥5	12695	644	17.84 (16.33-19.49)	9.38 (8.55-10.28)
Abbreviations: OR, odds ratio. Model 1 is a crude model (univariable). Model 2 is an adjusted model (multivariable), with adjustments for year of birth, county, and educational attainment.				

For peer review only

Supplementary Table S4. Odds ratio for VTE according to multimorbidity score (0 to ≥5) in females. Odds Ratios (ORs) with 95 % confidence interval (CI) for multimorbidity scores. Reference with no diseases (score=0).

	Female (n=1312989)			
	OR (95% CI)			
VTE score	No VTE	VTE	Model 1	Model 2
Score 0	728838	2499	1[Reference]	1[Reference]
Score 1	325513	2208	1.98 (1.88-2.10)	1.84 (1.73-1.94)
Score 2	140025	1463	3.05 (2.86-3.25)	2.67 (2.5-2.85)
Score 3	60906	902	4.32 (4.0-4.66)	3.53 (3.26-3.81)
Score 4	27081	580	6.25 (5.70-6.84)	4.76 (4.34-5.22)
Score ≥5	22015	959	12.70 (11.78-13.70)	8.58 (7.94-9.27)

Abbreviations: OR, odds ratio. Model 1 is a crude model (univariable). Model 2 is an adjusted model (multivariable), with adjustments for year of birth, county, and educational attainment.

For peer review only

Supplementary Table S5. Odds ratio for VTE according to multimorbidity score (0 to ≥5) for individuals born 1947-1972. Odds Ratios (ORs) with 95 % confidence interval (CI) for multimorbidity scores. Reference with no diseases (score=0).

	All (n=641211)			
	OR (95% CI)			
VTE score	NO VTE	VTE	Model 1	Model 2
Score 0	340353	2331	1[Reference]	1[Reference]
Score 1	156444	2062	1.92 (1.78-2.00)	1.89 (1.78-2.00)
Score 2	70005	1437	3 (2.81-3.2)	2.89 (2.7-3.08)
Score 3	33176	957	4.22 (3.9-4.54)	4 (3.7-4.31)
Score 4	16542	624	5.51 (5.04-6.02)	5.13 (4.68-5.62)
Score ≥5	16266	1014	9.1 (8.44-9.81)	8.3 (7.68-8.97)

Abbreviations: OR, odds ratio. Model 1 is a crude model (univariable). Model 2 is an adjusted model (multivariable), with adjustments for sex, year of birth, county, and educational attainment.

For peer review only

Supplementary Table S6. Odds ratio for VTE according to multimorbidity score (0 to ≥5) for individuals born 1972-1983. Odds Ratios (ORs) with 95 % confidence interval (CI) for multimorbidity scores. Reference with no diseases (score=0).

	All (n=682582)			
	OR (95% CI)			
VTE score	NO VTE	VTE	Model 1	Model 2
Score 0	415987	1638	1[Reference]	1[Reference]
Score 1	155822	1277	2.08 (1.93-2.24)	2.01 (1.87-2.16)
Score 2	60801	725	3.02 (2.77-3.31)	2.85 (2.61-3.11)
Score 3	25237	468	4.71 (4.25-5.22)	4.33 (3.9-4.81)
Score 4	11184	280	6.36 (5.59-7.23)	5.74 (5.04-6.54)
Score ≥5	8779	384	11.11 (9.92-12.44)	9.7 (8.64-10.89)

Abbreviations: OR, odds ratio. Model 1 is a crude model (univariable). Model 2 is an adjusted model (multivariable), with adjustments for sex, year of birth, county, and educational attainment.

For peer review only

Supplementary Table S7. Odds ratio for VTE according to multimorbidity score (0 to ≥5) for individuals born 1983-1993. Odds Ratios (ORs) with 95 % confidence interval (CI) for multimorbidity scores. Reference with no diseases (score=0).

	All (n=691267)			
	OR (95% CI)			
VTE score	NO VTE	VTE	Model 1	Model 2
Score 0	427804	845	1[Reference]	1[Reference]
Score 1	156178	624	2.02 (1.82-2.24)	1.94 (1.75-2.15)
Score 2	61537	399	3.28 (2.91-3.7)	3.04 (2.69-3.43)
Score 3	25768	204	4.01 (3.44-4.67)	3.59 (3.07-4.2)
Score 4	10734	137	6.46 (5.39-7.75)	5.63 (4.68-6.78)
Score ≥5	6858	179	13.21 (11.23-15.55)	10.91 (9.21-12.91)

Abbreviations: OR, odds ratio. Model 1 is a crude model (univariable). Model 2 is an adjusted model (multivariable), with adjustments for sex, year of birth, county, and educational attainment.

For peer review only - http://bmjopen.bmj.com/site/about/guidelines.xhtml

Supplementary Table S8. Odds ratio for VTE according to multimorbidity score (0 to ≥5) for individuals born 1993-2005. Odds Ratios (ORs) with 95 % confidence interval (CI) for multimorbidity scores. Reference with no diseases (score=0).

	All (n=679382)			
	OR (95% CI)			
VTE score	NO VTE	VTE	Model 1	Model 2
Score 0	427061	194	1[Reference]	1[Reference]
Score 1	164935	145	1.94 (1.56-2.4)	1.94 (1.56-2.41)
Score 2	59215	91	3.38 (2.64-4.34)	3.16 (2.46-4.06)
Score 3	18788	36	4.22 (2.95-6.02)	3.49 (2.44-5)
Score 4	6062	22	7.99 (5.14-12.43)	5.92 (3.8-9.24)
Score ≥5	2807	26	20.4 (13.52-30.76)	13.46 (8.87-20.42)

Abbreviations: OR, odds ratio. Model 1 is a crude model (univariable). Model 2 is an adjusted model (multivariable), with adjustments for sex, year of birth, county, and educational attainment.

For peer review only - http://bmjopen.bmj.com/site/about/guidelines.xhtml

STROBE 2007 (v4) Statement—Checklist of items that should be included in reports of *cross-sectional studies*

Section/Topic	Item #	Recommendation	Reported on page #
**Title and abstract**	1	(*a*) Indicate the study's design with a commonly used term in the title or the abstract	1-3
		(*b*) Provide in the abstract an informative and balanced summary of what was done and what was found	2-3
**Introduction**			
Background/rationale	2	Explain the scientific background and rationale for the investigation being reported	5-7
Objectives	3	State specific objectives, including any prespecified hypotheses	5-7
**Methods**			
Study design	4	Present key elements of study design early in the paper	7-8
Setting	5	Describe the setting, locations, and relevant dates, including periods of recruitment, exposure, follow-up, and data collection	7-8
Participants	6	(*a*) Give the eligibility criteria, and the sources and methods of selection of participants	7-8
Variables	7	Clearly define all outcomes, exposures, predictors, potential confounders, and effect modifiers. Give diagnostic criteria, if applicable	8-9
Data sources/ measurement	8*	For each variable of interest, give sources of data and details of methods of assessment (measurement). Describe comparability of assessment methods if there is more than one group	8-9
Bias	9	Describe any efforts to address potential sources of bias	*9-10*
Study size	10	Explain how the study size was arrived at	7-10
Quantitative variables	11	Explain how quantitative variables were handled in the analyses. If applicable, describe which groupings were chosen and why	7-10
Statistical methods	12	(*a*) Describe all statistical methods, including those used to control for confounding	9-10
		(*b*) Describe any methods used to examine subgroups and interactions	9-10
		(*c*) Explain how missing data were addressed	9-10
		(*d*) If applicable, describe analytical methods taking account of sampling strategy	9-10
		(*e*) Describe any sensitivity analyses	9-10
**Results**			

For peer review only

Participants	13*	(a) Report numbers of individuals at each stage of study—eg numbers potentially eligible, examined for eligibility, confirmed eligible, included in the study, completing follow-up, and analysed	11-17
		(b) Give reasons for non-participation at each stage	NA -nationwide study
		(c) Consider use of a flow diagram	Not used
Descriptive data	14*	(a) Give characteristics of study participants (eg demographic, clinical, social) and information on exposures and potential confounders	11-17
		(b) Indicate number of participants with missing data for each variable of interest	NA -nationwide study
Outcome data	15*	Report numbers of outcome events or summary measures	11-17
Main results	16	(*a*) Give unadjusted estimates and, if applicable, confounder-adjusted estimates and their precision (eg, 95% confidence interval). Make clear which confounders were adjusted for and why they were included	11-17
		(*b*) Report category boundaries when continuous variables were categorized	11-17
		(*c*) If relevant, consider translating estimates of relative risk into absolute risk for a meaningful time period	11-17
Other analyses	17	Report other analyses done—eg analyses of subgroups and interactions, and sensitivity analyses	11-17
**Discussion**			
Key results	18	Summarise key results with reference to study objectives	18-21
Limitations	19	Discuss limitations of the study, taking into account sources of potential bias or imprecision. Discuss both direction and magnitude of any potential bias	18-21
Interpretation	20	Give a cautious overall interpretation of results considering objectives, limitations, multiplicity of analyses, results from similar studies, and other relevant evidence	18-21
Generalisability	21	Discuss the generalisability (external validity) of the study results	18-21
**Other information**			
Funding	22	Give the source of funding and the role of the funders for the present study and, if applicable, for the original study on which the present article is based	21

*Give information separately for cases and controls in case-control studies and, if applicable, for exposed and unexposed groups in cohort and cross-sectional studies.

Note: An Explanation and Elaboration article discusses each checklist item and gives methodological background and published examples of transparent reporting. The STROBE checklist is best used in conjunction with this article (freely available on the Web sites of PLoS Medicine at http://www.plosmedicine.org/, Annals of Internal Medicine at http://www.annals.org/, and Epidemiology at http://www.epidem.com/). Information on the STROBE Initiative is available at www.strobe-statement.org.

For peer review only

For peer review only
